# Hua-Tan-Sheng-Jing Decoction Treats Obesity With Oligoasthenozoospermia by Up-Regulating the PI3K-AKT and Down-Regulating the JNK MAPK Signaling Pathways: At the Crossroad of Obesity and Oligoasthenozoospermia

**DOI:** 10.3389/fphar.2022.896434

**Published:** 2022-04-26

**Authors:** Yang Dong, Yanfei Zheng, Linghui Zhu, Tianxing Li, Yuanyuan Guan, Shipeng Zhao, Qi Wang, Ji Wang, Lingru Li

**Affiliations:** ^1^ School of Traditional Chinese Medicine, Beijing University of Chinese Medicine, Beijing, China; ^2^ National Institute of TCM Constitution and Preventive Medicine, Beijing University of Chinese Medicine, Beijing, China; ^3^ Institute of Basic Theory for Chinese Medicine, China Academy of Chinese Medical Sciences, Beijing, China; ^4^ Graduate School, China Academy of Chinese Medical Sciences, Beijing, China

**Keywords:** Hua-Tan-Sheng-Jing Decoction (HTSJD), network pharmacology, molecular docking, obesity, oligoasthenozoospermia, PI3K-AKT signaling pathway, MAPK signaling pathway

## Abstract

**Background:** Oligoasthenozoospermia is the leading cause of male infertility, seriously affecting men’s health and increasing the societal medical burden. In recent years, obesity-related oligoasthenozoospermia has attracted increased attention from researchers to find a cure. This study aimed to evaluate the efficacy of Hua-Tan-Sheng-Jing decoction (HTSJD) in treating obesity with oligoasthenozoospermia, determine its active ingredients and identify its mechanism of action.

**Methods:** The ingredients of HTSJD were determined by combining the ultra-performance liquid chromatography with mass spectrometry (UPLC-MS/MS) and systems pharmacology approach. The common pathogenesis of obesity and oligoasthenozoospermia and the potential mechanism of HTSJD against obesity with oligoasthenozoospermia were obtained through target fishing, network construction, and enrichment analyses. Further, molecular docking of the key ingredients with the upstream receptors of the key signaling pathways of the potential mechanism was used to predict their affinity. Finally, high-fat-induced obesity with oligoasthenozoospermia rat model was constructed to determine the effects of HTSJD on semen concentration, sperm motility, body weight, and serum lipid metabolism. The key proteins were validated by immunohistochemistry (IHC).

**Results:** A total of 70 effective components and 847 potential targets of HTSJD (H targets) were identified, of which 743 were common targets related to obesity and oligoasthenozoospermia (O-O targets) mainly enriched in the pathways related to inflammation, oxidative stress and hormone regulation. Finally, 143 common targets (H-O-O targets) for HTSJD against obesity with oligoasthenozoospermia were obtained. Combining the hub genes and the results of Gene Ontology (GO) functional and Kyoto Encyclopedia of Genes and Genomes (KEGG) pathway analysis of H-O-O targets, PI3K-AKT and MAPK signaling pathways were identified as the key pathways. Molecular docking results showed that Diosgenin, Kaempferol, Quercetin, Hederagenin, Isorhamnetin may act on the related pathways by docking EGFR, IGF1R and INSR. The animal-based *in vivo* experiments confirmed that HTSJD improves the sperm quality of high-fat diet-fed rats by reducing their body weight and blood lipid levels, influencing the PI3K-AKT and MAPK signaling pathways and altering the corresponding protein expressions.

**Conclusion:** HTSJD treats obesity with oligoasthenozoospermia by up-regulating the PI3K-AKT signaling pathway and down-regulating the MAPK signaling pathway, which are at the crossroad of obesity and oligoasthenozoospermia.

## Introduction

With the increase in the prevalence of unhealthy lifestyles such as excessive sugar and fat intake, reduced physical activity, and sedentary lifestyles, the obesity rate has rapidly increased in today’s society. Currently, nearly one-third of the world’s population is classified as overweight or obese, which is double the reports from 1980 (Chooi et al., 2019). In China, about 46% of the adults are either obese or overweight, making it the most affected country globally (Wang H. et al., 2017). Not only has obesity been associated with diabetes, cardiovascular disease, cancer, asthma, sleep disorders, and an increased risk of all-cause mortality (Flegal, 2017), but also infertility (Kahn and Brannigan, 2017; Liu and Ding, 2017). Specifically, obesity decreases semen quality, further affecting the reproductive capacity (Martins et al., 2018; Abdel-Fadeil et al., 2019; Crean and Senior, 2019). Thus, overweight men are more likely to have oligozoospermia or azoospermia compared to normal-weight men (Sermondade et al., 2013). Besides, the mean DNA fragmentation index (DFI) of sperms in obese men is significantly higher than in non-obese men, and it is significantly lowered following weight loss (Mir et al., 2018). Therefore, an early intervention against obesity or overweight in male populations suffering from infertility is crucial. However, even with several drugs against obesity being currently approved for marketing or in clinical trials, they are more often developed for weight loss in large weight groups; thus, their safety and efficacy are yet to be proven (Yanovski and Yanovski, 2021).

HTSJD is a clinical formula developed by Professor Wang Qi to treat obesity with oligoasthenozoospermia. Professor Wang is a well-known Chinese male medicine expert, awarded the highest honor (National Medical Master) in Chinese medicine. HTSJD is made by adding and subtracting Huatan Qushi decoction (HTQSD), which consisting of *Hedysarum Multijugum Maxim* (Huangqi), *Atractylodes Lancea* (Cangzhu), *Citrus Reticulata* (Chenpi), *Poria Cocos* (Fuling), *Alisma Orientale* (Zexie), *Coicis Semen* (Yiyiren), *Cinnanmomi Cortex* (Rougui), *Amomum villosum Lour* (Sharen), *Cyperi Rhizoma* (Xiangfu), *Plantago Asiatica L* (Cheqianzi), and *Polygonati Rhizoma* (Huangjing). The formula resolves phlegm, dispels dampness, benefits the kidney, and enhances sperm production. Previous studies have shown that HTQSD significantly reduces the body mass index (BMI) of obese people, improving lipid metabolism and their quality of life (Zhang et al., 2021). Besides, Huangqi, a monarch drug in HTSJD, lowers blood lipid and lipid peroxidation levels in hyperlipidemic rats (Wang et al., 2010; Cheng et al., 2011) and enhances the sperm count and viability (Kim et al., 2016). Additionally, Cheqianzi reduces high-fat diet-induced lipid accumulation (Yang et al., 2017). It is an important drug in Wuzi Yanzong Pill (WZYZP), a traditional spermatogenic drug that improves spermatogenic dysfunction in mice by modulating pro-inflammatory cytokine levels (Pan et al., 2021).

**FIGURE 1:**
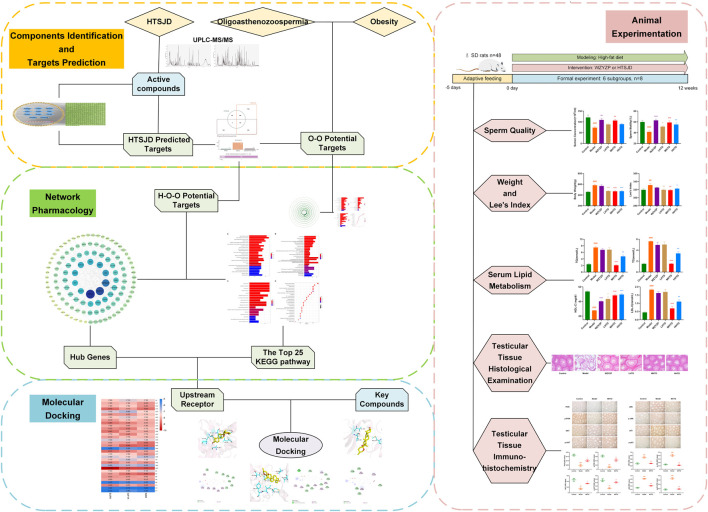
Flow chart for exploring the mechanism of obesity and oligoasthenozoospermia treatment by HTSJD.

Although clinical and fundamental studies have established the good effects of formulas and ingredients in herbal medicine, it has been impossible to identify their action mechanism due to the complexity of the mechanism of action of herbal medicines. Therefore, this study investigated the mechanism of action of HTSJD in the treatment of obesity-related complications such as oligoasthenozoospermia based on a combination of network pharmacology and molecular docking, combined with animal experiments (Figure 1). The findings in this study will provide a promising strategy for treating obesity with oligoasthenozoospermia, which is a contributing factor to male infertility. There is an increased risk of death in men who have male infertility than highly fertile men (Ventimiglia et al., 2015). Therefore, timely detection and intervention of male infertility can not only significantly improve male health, but also reduce social medical burden (Tvrda et al., 2015).

## Material and Methods

### Preparation of HTSJD

HTSJD was purchased from Beijing Tcmages Pharmaceutical Co., Ltd. (Beijing, China). The batch numbers for each herb are summarized in Supplementary Table S1.

### Ingredients Identification and Targets Prediction

#### Liquid Chromatographic and Mass Spectrometer Conditions

A total of 2.6 g of the Chinese medicine granule mixture was weighed, 10 ml of hot water added and brewed for 20 min. Next, the mixture was sonicated for 5 min followed by a 10min centrifugation at 12000 r/min. The supernatant was filtered through a 0.45 um membrane, and the filtrate was injected into a hybrid quadrupole time-of-flight liquid chromatography mass spectrometer, an ultra-high performance liquid chromatography (UPLC, Shimadzu LC-30) connected to a mass spectrometer (MS, SCIEX 5600+) with a Shimadzu InertSustain C18 column (100 × 2.1 mm, 2 µm). The column temperature was set at 35°C and 1 ml/min flow rate. The mobile phase consisted of acetonitrile (A) and 0.1% HCOOH-H2O (B).

Mass spectrometric detection was performed using electrospray ionization (ESI) in the positive and negative ion modes.

#### Collection of Active Ingredients and Corresponding Targets of HTSJD

The compounds in the 11 herbs composing HTSJD were obtained from the TCMSP (https://tcmsp-e.com/). The bioactive components were selected based on oral bioavailability and drug-likeness properties. The common screening criteria were oral bioavailability ≥20% and drug-likeness ≥0.1. The molecular ingredients identified by UPLC-MS/MS and the bioactive properties obtained from the TCMSP database collectively formed the active ingredients of HTSJD. The upset plot of HTSJD active ingredients was drawn using the TBtools software (Chen et al., 2020).

Next, the Swiss Target Prediction (Gfeller et al., 2014) and TCMSP were used to predict the potential targets for HTSJD bioactive ingredients. The identified potential protein targeted by the HTSJD bioactive ingredients was converted to a gene using the UniProt Knowledgebase (Uniprotconsortium, 2021).

Finally, the active ingredient-target protein network was constructed using the Cytoscape 3.8.2 (Shannon et al., 2003). Edges connected the nodes, and the HTSJD key active ingredients were obtained by screening the nodes with degree values greater than the median.

#### Collection of Known Targets Associated With Oligoasthenozoospermia-Obesity

Oligoasthenozoospermia and obesity were used as the search keywords from TTD (http://db.idrblab.net/ttd/), DisgenetGene (https://www.disgenet.org/), Drugbank database (https://www.drugbank.ca), and OMIM (http://omim.org/). Deduplication was performed after normalizing the targets numbers according to UniProt Knowledgebase.

#### Acquisition of HTSJD-Oligoasthenozoospermia-Obesity Common Targets

The common targets when treating oligoasthenozoospermia and obesity were obtained after the intersection of the HTSJD and diseases targets.

### Construction of Protein-Protein-Interactions Network and Screening of Hub Genes

The O-O and H-O-O targets were respectively submitted into the String (Szklarczyk et al., 2021) (https://string-db.org/) database to construct the protein interaction network. The species was limited to “Homo sapiens” and the confidence score was 0.9. The PPI interactive networks were then constructed and visualized using the Cytoscape 3.8.2 software.

Next, the Cytoscape plug-ins CytoHubba (Chin et al., 2014), and CytoNCA (Tang et al., 2015) were used to rank the H-O-O PPI network using the Maximum Neighboring Ingredients (MNC), Maximum Density of Neighboring Ingredients (DMNC), Maximum Creel Centrality (MCC), Edge Percolated Ingredient (EPC), Stress and Betweenness Centrality, Closeness Centrality, and Degree Centrality ranking. The targets screened using the two plug-ins were intersected to obtain the hub genes of HTSJD for obesity treatment.

### Gene Ontology Function and Kyoto Encyclopedia of Genes and Genomes Pathway Enrichment Analysis

The Gene Ontology (GO) enrichment analysis, including biological processes (BP), molecular function (MF) and cellular ingredients (CC), and Kyoto Encyclopedia of Genes and Genomes (KEGG) pathway enrichment analysis were performed using the R package (clusterProfiler) (Yu et al., 2012) to predict the mechanism of action. A *p*-value of 0.01 and a Q-value of 0.05 was used as the cut-off for gene radio using ggplot2 in R software. Finally, a herb-ingredient-target-pathway (H-I-T-P) network was constructed using the Cytoscape software.

### Molecular Docking

The 3D structure of each ingredient was retrieved from PubChem (https://pubchem.ncbi.nlm.nih.gov/), minimizing the energy using the Chem3D software. The protein crystal structures of macromolecular receptors were retrieved from the Uniprot/Protein Data Bank (PDB, https://www.rcsb.org/) and saved in PDB format. The water molecules and proligands were removed from the proteins using Pymol (Version 2.5.0, Schrödinger, LLC), and the proteins were modified by hydrogenation and charge balance using AutoDock Tools (Morris et al., 2009), then saved in pdbqt format. Next, the small molecule ligands and receptor proteins were docked using the QuickVina 2.1 software (Alhossary et al., 2015), and their binding potential was evaluated using an affinity score. A score less than -7 indicated that the ligand could spontaneously bind to the receptor.

The crystal structures of the downloaded receptor proteins were also re-docked before the formal molecular docking. Finally, the docking results were visualized using Pymol and Discovery Studio Standalone software.

### Animal Experimentation

#### Drug Preparation and Dosage

WZYZP, a Chinese medicine used to treat male infertility, was purchased from Beijing Tongrentang. Co. (Beijing, China; batch NO. Z11020188). The pills were ground into powder and dissolved in distilled water, and 11 g of the powder was added per 100 ml of water.

In HTSJD preparation, the Huangqi, Cangzhu, Chenpi, Fuling, Zexie, Yiyiren, Rougui, Sharen, Xiangfu, Cheqianzi, and Huangjing herbal granules were mixed in the ratio of 20:15:15:15:15:20:6:10:10:15 and dissolved in distilled water.

Dose calculations for the HTSJD and WZYZP were as follows.

$$D_{R}=\frac{D_{H}}{W}\times F$$

Where D_R_ is the drug dose used in this experiment, D_H_ is the clinical human drug dose, W is the human body weight, taken here as 70kg, and F is the dose conversion factor for rats and humans, calculated from the body surface area, taken as 6.3.

The final concentration of WZYZP suspension was 0.11 g/ml. Hua-Tan-Sheng-Jing decoction (HTSJD) was formulated into three concentrations: low, medium, and high, consisting of 0.66 g/ml, 1.32 g/ml, and 2.64 g/ml HTSJD, respectively. The medium concentration was the human equivalent dose, and the other two were half and twice the equivalent dosage, respectively.

#### Study Animals and Housing Conditions

Healthy, specific pathogen-free male Wistar rats (mass: 200 ± 20 g) were purchased from the Laboratory Animal Centre, Si Beifu, with a Laboratory Animal Certificate of Conformity (SCXC (Jing) 2016–0,002). Their housing conditions were: temperature, 24.0 ± 1.0 C; relative humidity, 55–65%; and a 12-h light/dark cycle. The rats were given *ad libitum* access to water and food. The experimental protocol was reviewed and approved by the Animal Care and Use Committee of Beijing University of Chinese Medicine of China (animal ethics number: BUCM-4–2,017,110,313).

#### Grouping and Model Establishment

After 5 days of adaptive feeding, 48 rats were assigned into six subgroups (*n* = 8), namely, the control group (C), model group (M), WZYZP group (W), low-dose HTSJD group (LHTD), medium-dose HTSJD group (MHTD) and high-dose HTSJD group (HHTD) using the random number table method.

The experimental period was 12 weeks, with modeling of the high-fat diet and gavage intervention performed simultaneously.

The control group was reared on a standard diet, while the other five groups were reared on a high-fat diet (Shengmin, Nanjing, China, formulated using the standard material, 18% lard, 18% fish meal, 15% sugar, 2% cholesterol and 0.2% bile salt). Groups C and M were gavaged with distilled water group W was intragastrically administered with WZYZP suspension, while groups LHTD, MHTD, and HHTD groups were intragastrically administered with low, medium, and high concentrations of HTSJD, respectively. The intragastric volume per rat was 10 ml/kg per day.

#### Body Weight and Lee’s Index Determination

Each rat was weighed every 7 days during the experiment. At the end of the experiment, rats were narcotized by 1% pentobarbital, and their body length (from nasal to caudal tip) was measured. Lee’s index was calculated to evaluate the degree of obesity:

$$Lee^{\prime }s\;index=\frac{Body\;Weight}{Body\;Length}$$

#### Determination of Biochemical Indexes

Serum samples were collected into plain tubes using the abdominal aortic method. The concentrations of HDL-C (mm-0667R1), LDL-C (mm-20864R1), TG (mm-0610R1), and TC (mm-0611R1) in serum were measured using ELISA kits (Jiangsu Meimian Industrial Co., Ltd., China).

#### Sperm Count and Motility Measurement

The rat’s cauda epididymis was minced in 500 µL phosphate-buffered saline (pH 7.2) and incubated for 5 min at 37°C to allow sperm release. Ten microliters of sperm suspension was added to the pre-warmed sperm counting plate, and the WL-9000 (WeiLi, Beijing, China) sperm quality testing system was used for analysis. Five fields of view were selected and the test was completed within 2 min.

#### Histological and Immune-Histological Examination

One rat testis per rat was removed and fixed in Bouin’s solution for 24 h. The testes were then dehydrated in graded ethanol, embedded in paraffin, and sectioned (4 µm in thicknesses). The sections were stained with hematoxylin-eosin (HE) dye and observed under a light microscope (Leica DM500, 100x).

Next, the sectioned paraffin-embedded slides of rats’ spermary per group were deparaffinized and rehydrated with xylene and gradient alcohol. The slides were then incubated with primary antibodies [PI3K p85 (60225-1-Ig, Proteintech), p-PI3K p85 (AF3242, Affinity), AKT (60203-2-Ig, Proteintech), p-AKT (AF0832, Affinity), JNK (66210-1-Ig, Proteintech), p-JNK (AF3318, Affinity), p53 (60283-2-Ig, Proteintech), and p-p53 (AF3075, Affinity)] overnight at 4°C following an antigen repair and closure. Next, the slides were incubated with a secondary antibody labeled with horseradish peroxidase (HRP) at room temperature for 1 h. The slides were then treated with 3,3-diaminobenzidine (DAB) and hematoxylin, dehydrated with alcohol, and sealed with neutral gum. Finally, slides’ images were captured by the Olympus BX53 fluorescence microscope (40x).

#### Statistical Analysis

All statistical analyses were performed using the SPSS software (version 26.0; SPSS, Chicago, United States). The differences between groups were analyzed using a one-way analysis of variance (ANOVA), and the means were compared by Dunnett’s *t*-test or Tamhane’s T2-test. Data were presented as mean ± standard error of the mean (SEM). *p* < 0.05 was considered statistically significant. All results were presented using GraphPad Prism 9 software.

## Results

### Identification of Active Ingredients and Predicted HTSJD Targets

**FIGURE 2:**
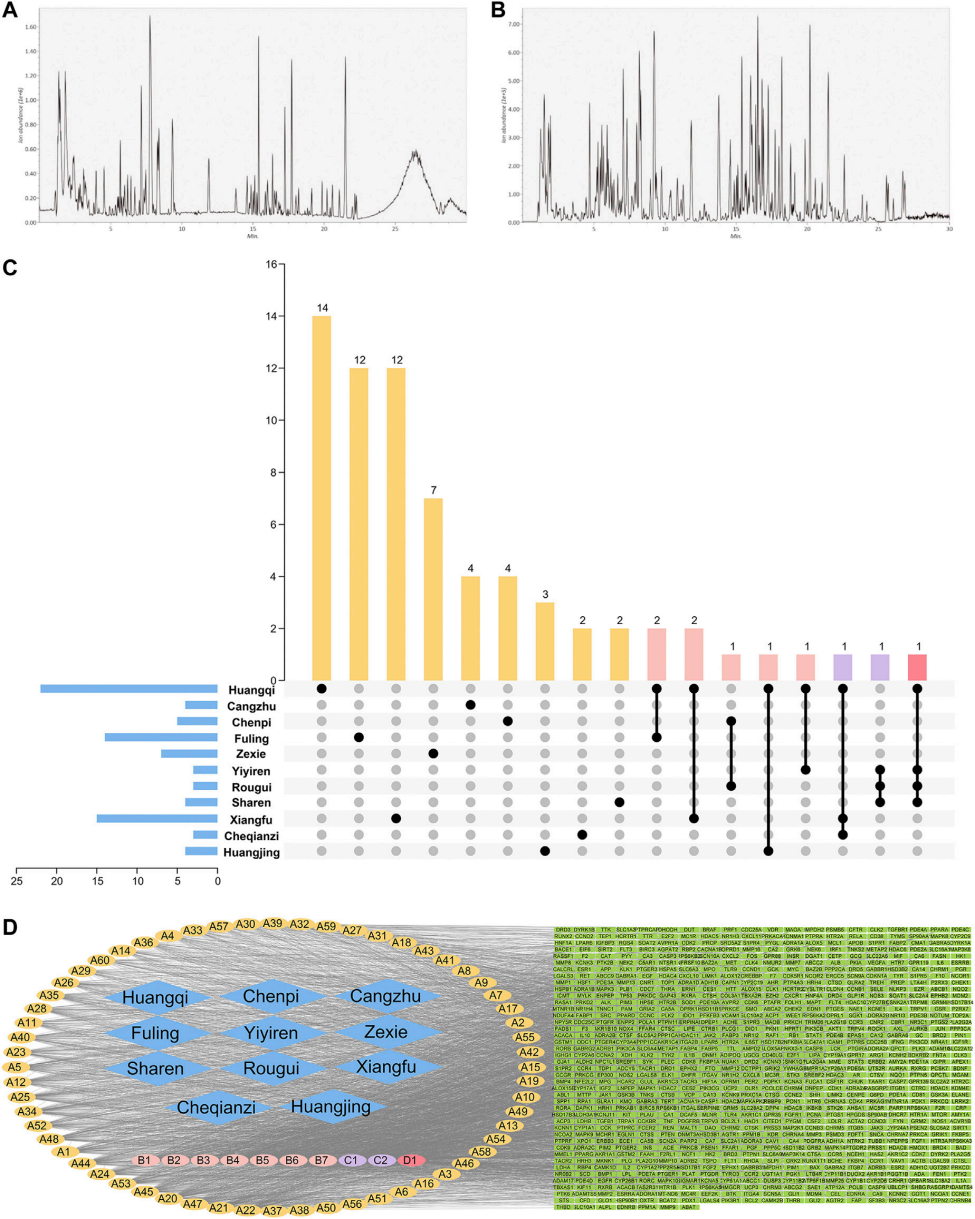
Positive ion **(A)** and negative ion **(B)** UPLC-MS/MS chromatography of HTSJD. **(C)** The number of unique and shared ingredients of each HTSJD herb. **(D)** The HTSJD ingredient-target visual network. Diamond nodes represent herbal medicines, oval nodes represent compounds, and square nodes represent targets. Compounds appearing in only one drug are highlighted yellow and denoted **(A)**. Compounds that appeared in two drugs are highlighted pink and denoted **(B)**. **(C,D)** represent common ingredients in three and four drugs, purple and red, respectively. The edges indicate interactions between compounds and the targets.

The UPLC-MS/MS system identified 370 compounds (Figures 2A,B), including 129 compounds in the positive ion mode, three in the negative ion mode, and 198 in positive and negative ion modes (Supplementary Tables S2 and S3).

After excluding duplicates, the database identified 203 bioactive ingredients from the HTSJD. Traditional Chinese medicine (TCM) prescriptions have thousands of ingredients, but only a few ingredients have satisfactory pharmacokinetics and pharmacodynamic characteristics that ultimately determine efficacy. The UPLC-MS/MS obtained molecules were compared with database molecules to identify corresponding ingredients. Non-corresponding molecules were eliminated, resulting in 70 HTSJD active ingredients. The 70 active ingredients were ranked by degree value. Ingredients with >41.5 median values were considered as key active ingredients. Therefore, the study identified 33 key active ingredients with corresponding Chinese herbs (Supplementary Table S4). Figure 2C shows the number of unique and common ingredients of each Chinese medicine.

After screening and de-duplication, we obtained 847 potential targets of the 70 bioactive ingredients and mapped them to human homologous genes using the UniProt database. The constructed herb-ingredient-target network (Figure 2D) had 928 nodes and 4,083 edges. The larger the value of the degrees, the more important the nodes are. Among the HTSJD ingredients, quercetin and kaempferol had the highest degree values (226 and 154, respectively).

### Collecting O-O Targets and Predicting Common Pathogenesis

**FIGURE 3:**
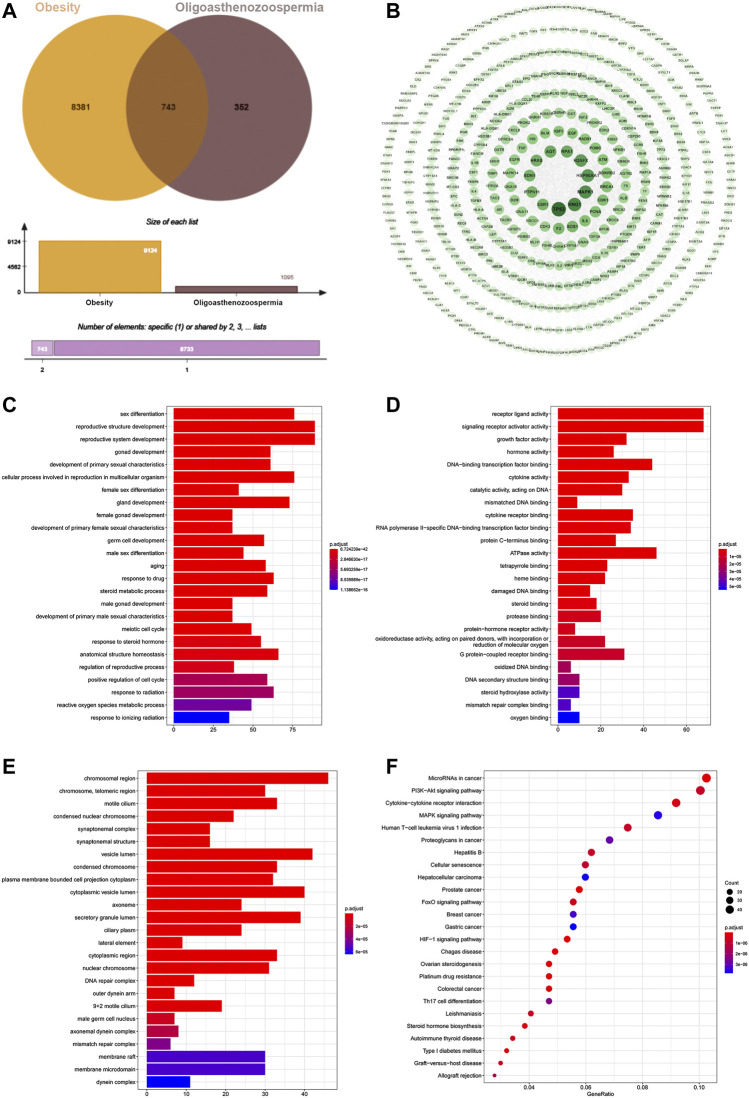
**(A)** Venn diagram of 743 target intersections for O-O. **(B)** The PPI network of O-O. The darker node color and larger font size represent larger degree values. **(C)** BP of the top 25 enriched GO terms and pathways. **(D)** MF of the top 25 enriched GO terms and KEGG pathways. **(E)** CC enrichment results of the top 25 enriched GO terms and KEGG pathways. **(F)** KEGG pathway of the top 25 and its corresponding target.

Pooling data from several databases, the target numbers for obesity, oligoasthenozoospermia and O-O were obtained as 1,095, 9,124 and 743, respectively (Figure 3A).

The O-O target data of protein-protein interaction from the String was imported into the Cytoscape software, which generated a PPI network with 514 nodes and 2,278 edges (Figure 3B). TP53, KNG1, MAPK1, HSP90AA1, and H2AFX were the top five proteins ranked by the node degree values.

The 743 O-O common targets were analyzed for GO functional and KEGG pathway enrichment using clusterProfiler, and the p. adjust-values and target counts ranked the results. Consequently, 1933 enriched GO functional terms were identified, including 1784 biological processes (BP), 81 molecular functions (MF), and 68 cellular components (CC). Figures 3C–E shows bar and bubble plots of the top 25 enriched GO functions and KEGG pathways, respectively. The GO functions related to obesity and oligoasthenozoospermia include reactive oxygen species metabolic process, germ cell development, hormone activity, oxidoreductase activity, synaptonemal structure, and male germ cell nucleus, and others. The top 25 pathways included the HIF-1 signaling pathway, FoxO pathway, PI3K-AKT signaling pathway, MAPK pathway, and Steroid hormone biosynthesis (Figure 3F). Indeed, the GO and KEGG enrichment results showed the common mechanisms between the two diseases.

### Network Pharmacological Analysis of the HTSJD Action Mechanism

**FIGURE 4:**
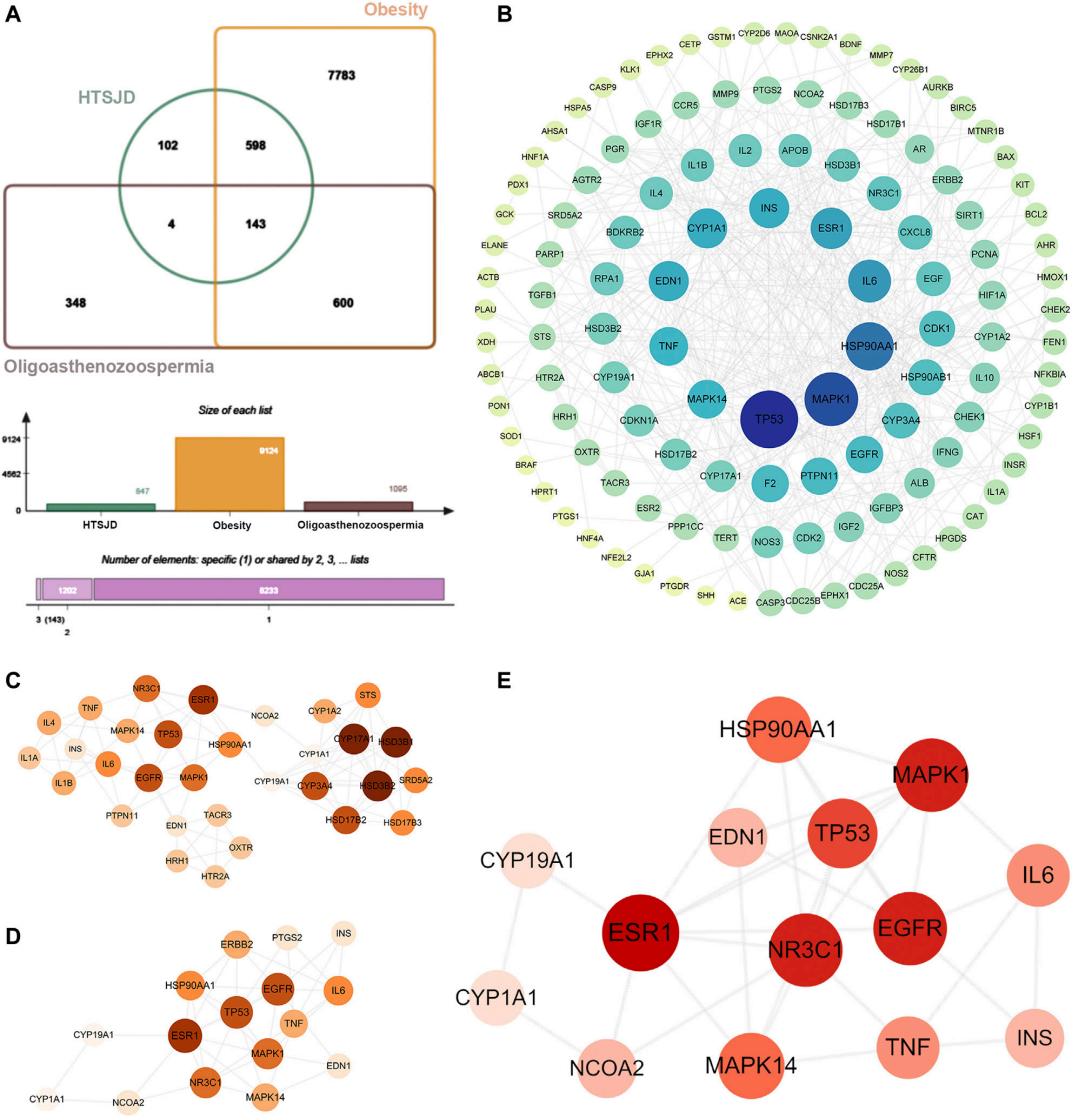
**(A)** Venn diagram showing 143 common H-O-O targets. **(B)** The PPI network of H-O-O. **(C)** Genes identified by the cytohubba. **(D)** Genes identified by the cytoNCA. **(E)** Hub genes were determined after the intersection. The darker node color or larger font size implies larger degree values.

The intersection of 847 drug targets with the above O-O targets generated 143 H-O-O common targets (Figure 4A). The protein-protein interaction network of the 143 H-O-O targets has 120 nodes and 428 edges (Figure 4B). The cytoHubba plugin of the PPI network proteins was sorted by the five different attributes and the top ten genes, yielding 31 targets after merging (Figure 4C). Similarly, the cytoNCA plugin generated 16 targets (Figure 4D). The intersection of the two network targets revealed 14 hub genes, namely TP53, HSP90AA1, MAPK1, IL6, ESR1, TNF, CYP1A1, EDN1, INS, CYP19A1, EGFR, MAPK14, NR3C1, and NCOA2 (Figure 4E).

**FIGURE 5:**
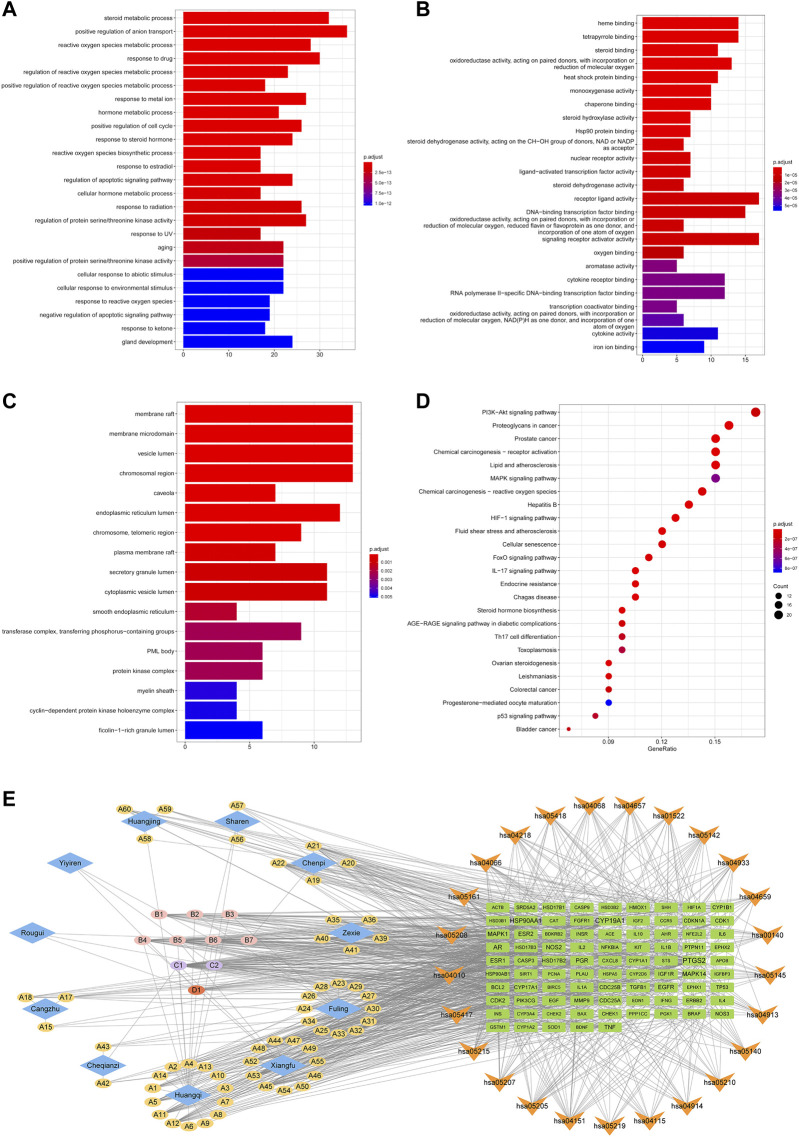
GO and KEGG pathway enrichment results of H-O-O common targets. **(A)** Results of BP enrichment analysis for top 20. **(B)** Results of MF enrichment analysis for top 20. **(C)** Results of CC enrichment analysis for top 17. **(D)** The top 25 KEGG pathways enriched by ingredients and corresponding targets. **(E)** Herb-ingredient-target-pathway (H-I-T-P) network of the HTSJD mechanism for treating O-O. The diamond represents the herbal medicine, the ellipse represents the active ingredient, the square represents the target, and the inverted triangle represents the KEGG pathway. The larger the label size, the larger the degree.

The GO functional and KEGG pathway enrichment analyses were performed on the 143 H-O-O common targets to further explore the mechanism of HTSJD action using the clusterProfiler R package. Figures 5A–D shows the top 25 GO functions and KEGG pathways. The results suggest that HTSJD probably acts in cellular ingredients such as membrane raft, vesicle lumen, chromosomal region, and the protein kinase complex. The active ingredients clustered into biological processes, including steroid metabolic process, reactive oxygen species metabolic process, hormone metabolic process, response to steroid hormone, and regulation of protein serine/threonine kinase activity. The enriched molecular functions include steroid binding, nuclear receptor activity, ligand-activated transcription factor activity, oxidoreductase activity, and signaling receptor activator activity. The KEGG enrichment analysis identified 121 pathways enriched by HTSJD against O-O, including the PI3K-AKT signaling pathway, lipid and atherosclerosis, MAPK signaling pathway, and steroid hormone biosynthesis, etc. Figure 5D shows the top 25 pathways selected and sorted according to the gene radio. The targets, ingredients, and herbs corresponding to these 25 pathways are displayed as network diagrams (Figure 5E).

**FIGURE 6:**
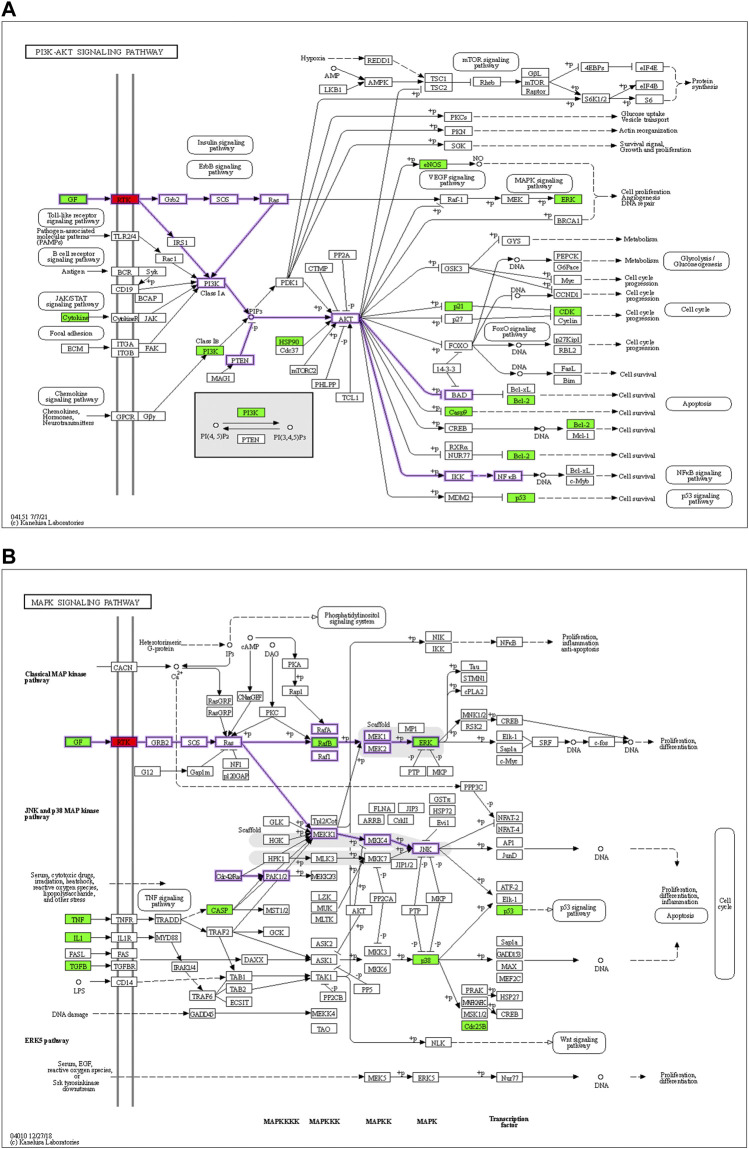
Diagrams of the PI3K-AKT signaling pathways **(A)** and MAPK signaling pathways **(B)**. Red means the docking target to be validated, and green represents the predicted drug action target of the net drug. The membrane RTK is a collective class of targets, including EGFR, INSR, and IGF1R.

Based on the identified hub genes and enrichment results of the H-O-O targets, we focused on the PI3K-AKT and MAPK signaling pathways. The mechanism of HTSJD treatment against obesity with oligoasthenozoospermia by targeting PI3K-AKT and MAPK signaling pathways is demonstrated in Figures 6A,B.

### Molecular Docking

Molecular docking of the key ingredients with the upstream receptors of the PI3K-AKT and MAPK signaling pathways was used to predict their affinity.

**FIGURE 7:**
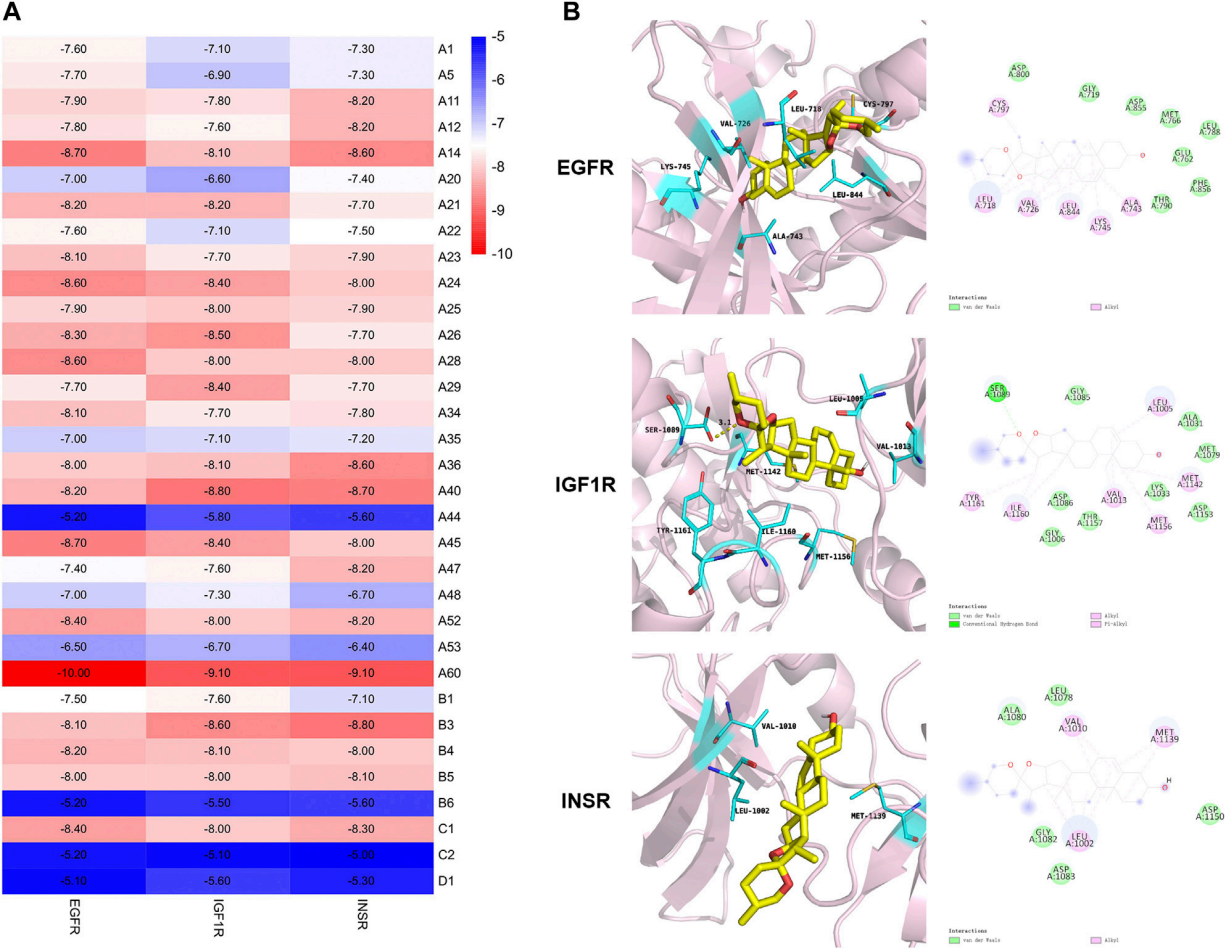
Molecular docking based on the binding affinity. **(A)** Heat map showing the affinity. **(B)** Stereoviews and binding poses of diosgenin (A60) optimal conformation docked to EGFR, IGF1R, and INSR.

The protein crystal structures (Table 1) were subjected to re-docking before the formal docking, and all the RMSDs were <2 Å (Supplementary Table S5), indicating that the docking methods and tools were well adapted to the conformations of these proteins (Cui et al., 2020). The docking results are displayed according to affinity (Figure 7A). A negative affinity score indicates that the ligand-acceptor docking does not require an external energy supply, while a lower affinity score indicates a higher probability of successful docking. The affinities of 99 docking results showed that the affinity of three proteins (3.03%) were ≤−9 kcal/mol, 78 (78.79%) were −9 to −7 kcal/mol, and 18 (18.18%) were > −7 but < −5 kcal/mol. Therefore, we considered affinities of ≤ −9 as the best docking conformation. We speculate that diosgenin plays a corresponding therapeutic role by binding to these three receptors and altering the expression of molecules downstream of the pathway.

**TABLE 1:** The protein crystal structure of key macromolecular receptors.

Gene	Identifier	Method	Resolution	Chain	Positions
EGFR	4LQM	X-ray	2.50 Å	A	694–1,022
IGF1R	5FXS	X-ray	1.90 Å	A	980–1,286
INSR	3EKK	X-ray	2.10 Å	A	1,005–1,310

The best conformation was further analyzed to demonstrate the binding mode. Figure 7B depicts the binding interactions of EGFR, IGF1R, and INSR with diosgenin according to the docking structures. The results show that hydrophobic H-bond interactions and other non-covalent interactions have key roles in influencing the binding affinity of protein complexes. In detail, diosgenin anchored to a hydrophobic pocket in EGFR and electrostatically interacted with Leu718, Val726, Ala743, Lys745, Cys797, and Leu844. In the IGF1R and INSR combination mode, these electrostatic interactions are key in influencing protein binding affinity. Moreover, the diosgenin structure also electrostatically interacted with Leu1005, Val1013, Met1142, Met1156, Ile1160, and Tyr1161 in IGF1R. The chemical structure of the hydroxyl hydrogen formed H-bond interactions with Ser1089. In INSR, the diosgenin structure had an electrostatic interaction with Leu1002, Val1010, and Met1139. Altogether, these interactions and docking energy show that the substrate binds tightly to amino acids deep in the cavity, revealing that small molecules are well suited to the receptor binding pockets.

### *In vivo* Assay

**FIGURE 8:**
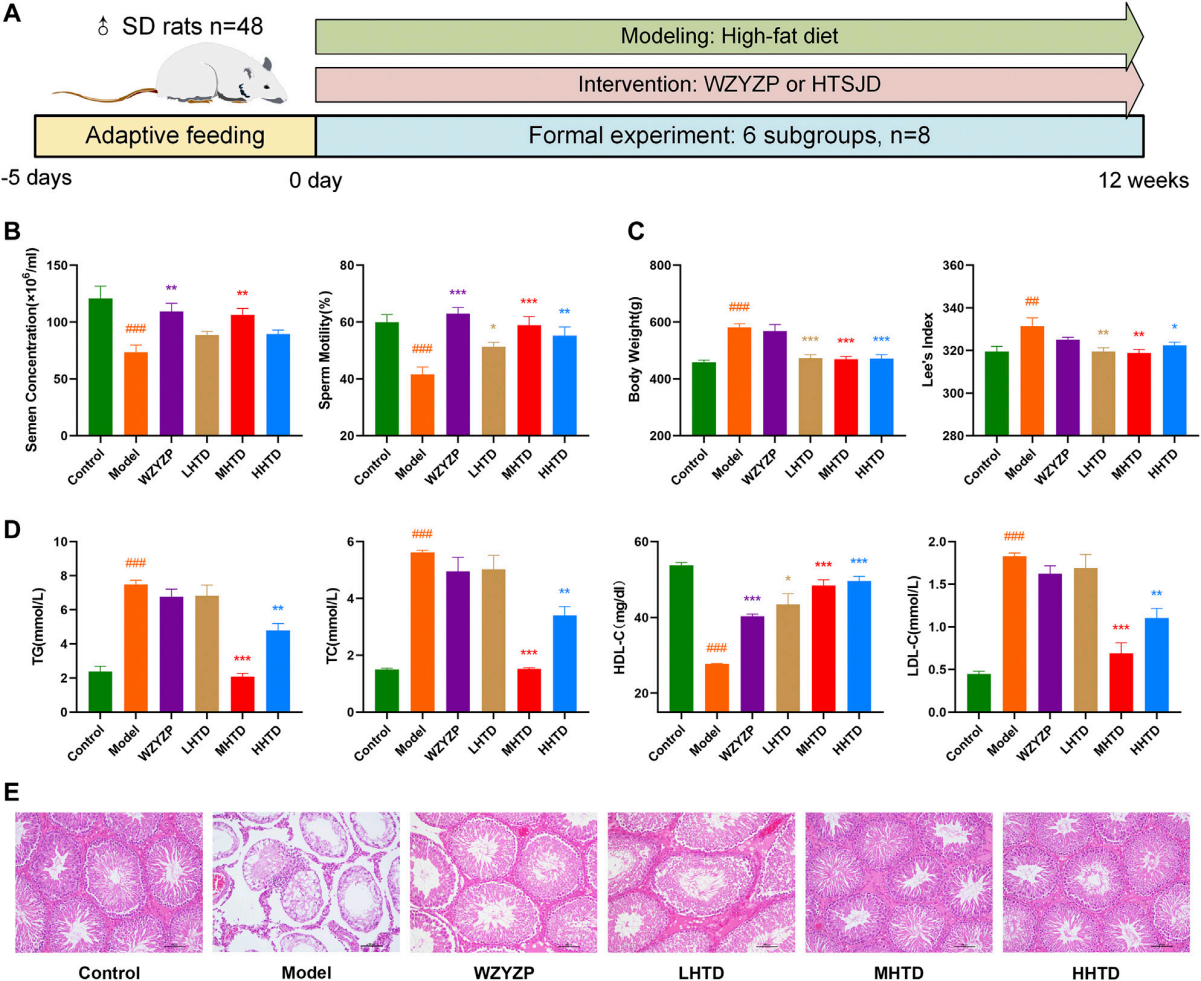
**(A)** Schematic presentation of the experiment design. **(B)** The effect of HTSJD on sperm quality. **(C)** The effect of HTSJD on body weight. **(D)** The effect of HTSJD on serum lipid metabolism. Notes: #*p* < 0.05, ##*p* < 0.01, ###*p* < 0.001 vs. control group; *p < 0.05, **p < 0.01, ***p < 0.001 vs. model group. **(E)** Histopathological examination of rat testes at ×100 magnification.

We established a rat model of obesity with oligoasthenospermia by high-fat diet feeding (Figure 8A). After 12 weeks, semen concentrations and sperm motility in the model group were lower than the control group (*p* < 0.001, Figure 8B). Besides, the body weight and Lee’s index were higher than the control group (*p* < 0.001, *p* < 0.01, Figure 8C), indicating that the oligoasthenozoospermia model was successfully established using the high-fat diet. HTSJD groups demonstrated a protective effect on sperm quality and reduced obesity, and the MHTD group showed the best results. Similar results were reported in the four items of blood lipid. Figure 8D shows the difference between the model and control group (*p* < 0.001). MHTD improved the disorder indicators caused by the high-fat diet (*p* < 0.001) and substantially normalized the indicators.

The testis germinal tubules of some rats in the model group showed structural changes (Figure 8E). The changes included degeneration and atrophy of the germinal tubules, thinning of the germinal epithelium, reduced number of spermatogenic cells in the tubule lumen, reduced interstitium, and some cells fell off into the tubule lumen. Infiltration of inflammatory cells was also observed. In contrast, all the spermatogenic cells in the testicular spermatogenic tubules of rats in the MHTD group were neatly and orderly arranged. Therefore, HTSJD possibly maintains normal germ cell morphology, protects the stability of spermatogenic structures, and prevents inflammatory cell infiltration.

**FIGURE 9:**
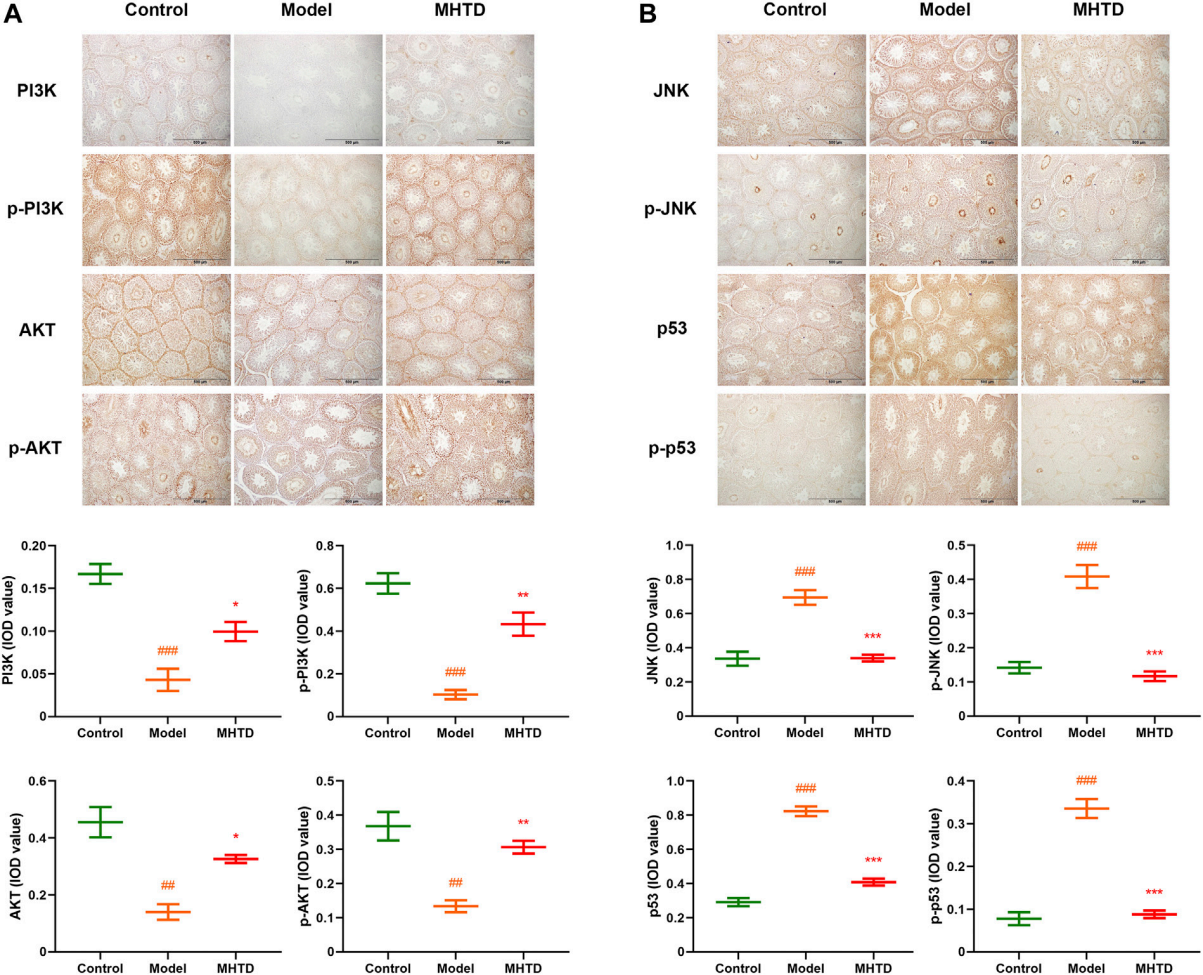
Immunohistochemical expression of PI3K, p-PI3K, AKT, p-AKT, JNK, p-JNK, p53, and p-p53 proteins. Notes: ###*p* < 0.001 vs. control group; *p < 0.05, **p < 0.01, ***p < 0.001 vs. model group.

We examined the expression and phosphorylation levels of important proteins on relevant pathways based on network pharmacological enrichment analysis. The aim was to investigate the mechanism of HTSJD intervention in obesity-induced oligoasthenozoospermia. The relative expression of PI3K/p-PI3K and AKT/p-AKT in the model group was lower than the control group (Figure 9). Moreover, the relative amounts of JNK/p-JNK and p53/p-p53 were higher in the model than in the control group. HTSJD controlled the indexes within a relatively normal range, significantly different from the model group. Thus, HTSJD probably activated the PI3K-AKT and JNK MAPK signaling pathways *in vivo*, inhibiting the obesity and sperm quality decline caused by the high-fat diet.

## Discussion

Oligoasthenozoospermia consists of oligozoospermia and asthenozoospermia and is the main cause of male infertility (Mehra et al., 2018). Abnormal spermatogenesis is caused by varicoceles, testicular injury, and orchitis, which change the testicular environment leading to abnormal spermatogenesis. In recent years, the relationship between obesity and male infertility has attracted much attention (Lainez and Coss, 2019). Obesity is a low-grade chronic inflammatory state accompanied by oxidative stress (Heilbronn and Campbell, 2008; Engin, 2017; Li et al., 2018; Li et al., 2020). In the testis, excessive accumulation of fat releases inflammatory factors, reactive oxygen species (ROS), and oxidative free radicals, which change the testis’ internal environment affecting the endocrine function of testicular tissues or disrupting the testicular tissue structure and sperm, directly leading to male infertility (Agarwal et al., 2003; Villegas et al., 2003; Bachir and Jarvi, 2014). In the present study, the HIF-1 signaling pathway, FoxO pathway, PI3K-AKT signaling pathway, MAPK pathway, and steroid hormone biosynthesis associated with inflammation, oxidative stress apoptosis, and hormone regulation were the common targets of oligoasthenozoospermia with obesity. These findings are consistent with previous literature reports (Kelly and Jones, 2015; Huang et al., 2016; Gonzalez et al., 2018; Cheng, 2019), which laid a foundation for this follow-up study on the action mechanism of HTSJD in treating obesity and oligoasthenozoospermia.

Based on traditional Chinese medicine, obese individuals prefer fatty, sweet, thick and greasy, less active, and sedentary habits, which are the pathogenesis factors in phlegm-dampness accumulation leading to a decline in sperm quality (Dong et al., 2018). HTSJD resolves the phlegm and dispelling dampness improving the bodys’ internal environment, which enhances the sperm quality when combined with drugs that benefit the kidney and sperm production. In the present study, the obesity and oligoasthenozoospermia model was established in rats (Suleiman et al., 2020) by feeding them a high-fat diet. As expected, the rats gained bodyweight with serum lipid metabolism disorder, testicular structure disorder, and a reduction in sperm quality. Our findings confirmed that HTSJD could reduce the weight gain and obesity degree in high-fat obese rats, maintain normal blood lipid levels, and improve the cellular morphology in the structural domain of the testis and sperm quality.

Given the complexity of Chinese herbal formulae, we explored the action mechanisms and active ingredients of HTSJD for treating obesity with oligoasthenozoospermia using UPLC-MS/MS, network pharmacology, molecular docking, and animal experiments. Our main focus was on the HTSJD mechanism on weight loss and sperm quality maintenance; thus, the common target against obesity and oligoasthenozoospermia (O-O targets) were used as the basis for the work. Finally, 143 H-O-O common targets were obtained. According to the PPI analysis on H-O-O targets, 14 hub genes were obtained. Combining the hub genes and the results on GO function and KEGG enrichment analysis, PI3K-AKT and MAPK signaling pathways were identified as the key pathways. PI3K and MAPK signaling pathways are major post-receptor cell signal transduction pathways in EGFR, IGF1R, and INSR (Zhang et al., 2014; Wee and Wang, 2017; Ju et al., 2019; Soza-Ried et al., 2019). They respond to extracellular inflammation, oxidative stress, insulin resistance, heat shock, and other stimuli, regulating a series of physiological and pathological activities, such as inflammation, oxidative stress, insulin resistance (IR), cellular intermediary metabolism, apoptosis, growth processes, and mitoses (Lebovitz, 2001; Cui et al., 2018; Wu et al., 2018; Simsek et al., 2021; Wu et al., 2021). Based on molecular docking, Diosgenin, Kaempferol, Quercetin, Hederagenin, and Isorhamnetin in HTSJD active ingredients regulates the expression of PI3K and MAPK signaling pathway by binding to EGFR, IGF1R, INSR. Quercetin inhibits EGFR phosphorylation, EGFR expression (Cuevas et al., 2011), the expression of related oxidative stress markers, and enhances GLUT4 transport in epididymal adipose tissue (Dong et al., 2014; Zhao et al., 2017). Diosgenin has a strong affinity in molecular docking, which plays a more significant role in the treatment process. It reduces the serum-free fatty acids and IL-6 levels, attenuates inflammatory responses associated with high-fat diets (Tharaheswari et al., 2014), and alleviates the testicular damage (Khosravi et al., 2019). Kaempferol reduces oxidative stress and inflammatory responses by downregulating the MAPK signaling pathways (Liu et al., 2021), inhibiting the expression of related oxidative stress markers, and enhancing GLUT4 transport in the epididymal adipose tissue (Jin et al., 2021). Hederagenin and Isorhamnetin reduce oxidative stress (Wang et al., 2020; Li et al., 2021). In conclusion, our findings were valid based on the network pharmacology and molecular docking, informing an *in vivo* validation.

A combination of drug targets and immunohistochemical techniques were used to validate the expression and phosphorylation of PI3K, AKT, JNK, and p53, and the key proteins in the PI3K-AKT and JNK MAPK signaling pathways in the testicular tissues of model and drug groups. Our results revealed that PI3K and AKT protein expression and phosphorylation in rat testis were significantly up-regulated, while JNK and p53 were significantly down-regulated, in the drug groups compared to the model group. PI3K-AKT and MAPK signaling pathways are key in obesity and spermatogenesis inhibition (Schultze et al., 2012; Huang et al., 2018; Eid et al., 2019; Ni et al., 2019). Obesity-related chronic inflammation, oxidative stress, mitochondrial dysfunction, endocrine stress, and metabolic disorder in animals induce IR through the PI3K-AKT pathway, which further leads to obesity aggravation, IR deterioration, and related metabolic disorders (Barber et al., 2021). However, the up-regulation of PI3K/AKT induces resistance against oxidative stress, improves lipid metabolism, and regulates dyslipidemia (Ajala-Lawal et al., 2020). At the same time, the inhibition of PI3K/AKT phosphorylation is closely associated with apoptosis in testis-supporting cells (Wang Y. et al., 2017). The JNK pathway in the MAPK pathway also bridges obesity and cell stress. The JNK pathway activated by obesity-related inflammation and oxidative stress initiates downstream cell differentiation, proliferation, and apoptosis effects (Solinas and Becattini, 2017). It mediates processes such as germ cell proliferation and apoptosis (Pinsino et al., 2015; Lu et al., 2021). The down-regulation of JNK in the MAPK pathway and the inhibition of p53 expression regulate the downstream target genes, thus alleviating oligoasthenozoospermia (Qi et al., 2014; Liu et al., 2015). This is consistent with our experimental verification results. The effect of HTSJD on sperm quantity and quality in obese rats may be due to the activated PI3K-AKT signaling pathway and down-regulated JNK MAPK signaling pathway to inhibit p53, which are at the crossroad of obesity and oligoasthenozoospermia.

Overall, our findings confirmed the relationship between obesity and sperm quality. Besides, the bioinformatics and molecular biology analysis revealed the efficacy and mechanism of HTSJD in treating obesity with oligoasthenozoospermia. The underlying mechanism entails resolving phlegm and dispelling dampness, benefiting the kidney and promoting sperm production. This study explores the common target of obesity and oligoasthenozoospermia as the entry point for in-depth research, providing a new research idea on their pharmacological mechanism. Network pharmacology and molecular docking are effective and reliable methods for studying multi-compound, multi-target drugs such as Chinese medicinal compounds. However, this method has limitations, including excessive reliance on database and software analysis, which may deviate the actual results due to imperfect database or software algorithms. Therefore, collecting data from as many databases as possible is the key to bringing the results closer to the real situation. This experiment integrated animal and molecular biology experiments based on information biology, which validated the data. In the follow-up experiments, clinical trials and cellular experiments are needed to explore the corresponding efficacy and mechanisms.

## Conclusion

HTSJD treats high-fat diet-induced obesity and oligospermia by activating the PI3K-AKT signaling pathway and inhibiting the JNK MAPK signaling pathway, which alleviates obesity and blood lipids, increasing sperm motility and semen concentration, preventing inflammatory infiltration within the testes.

## Data Availability

The original contributions presented in the study are included in the article/Supplementary Material, further inquiries can be directed to the corresponding authors.
