# Supplementary material for: Hua-Tan-Sheng-Jing Decoction Treats Obesity With Oligoasthenozoospermia by Up-Regulating the PI3K-AKT and Down-Regulating the JNK MAPK Signaling Pathways: At the Crossroad of Obesity and Oligoasthenozoospermia
Source: Front Pharmacol. 2022 Apr 26;13:896434. doi: 10.3389/fphar.2022.896434 (PMC9086321; doi:10.3389/fphar.2022.896434)
Supplement: Supplementary file 1 [file Presentation1.PPTX]

## Slide 1
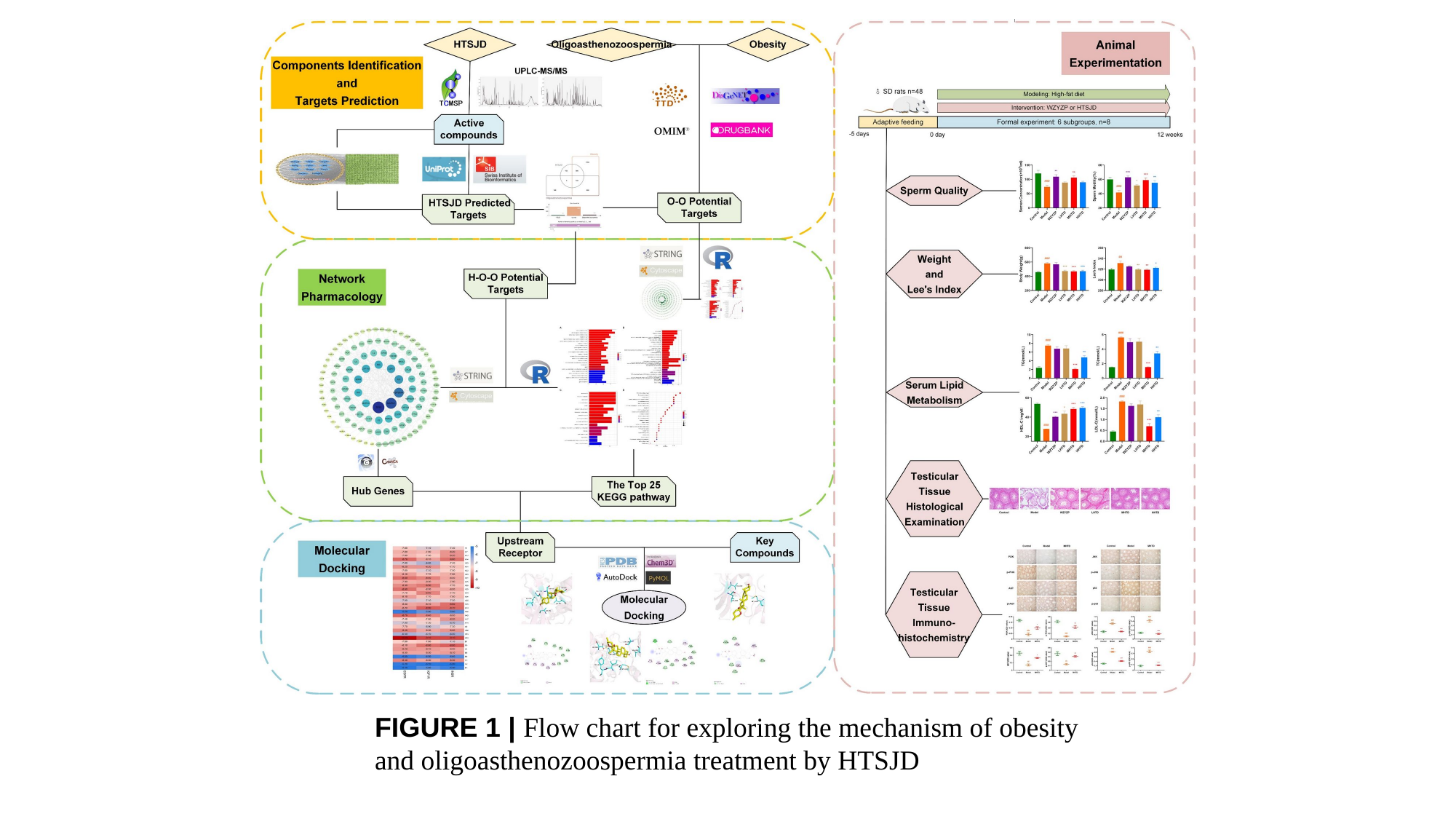

FIGURE 1 | Flow chart for exploring the mechanism of obesity and oligoasthenozoospermia treatment by HTSJD

## Slide 2
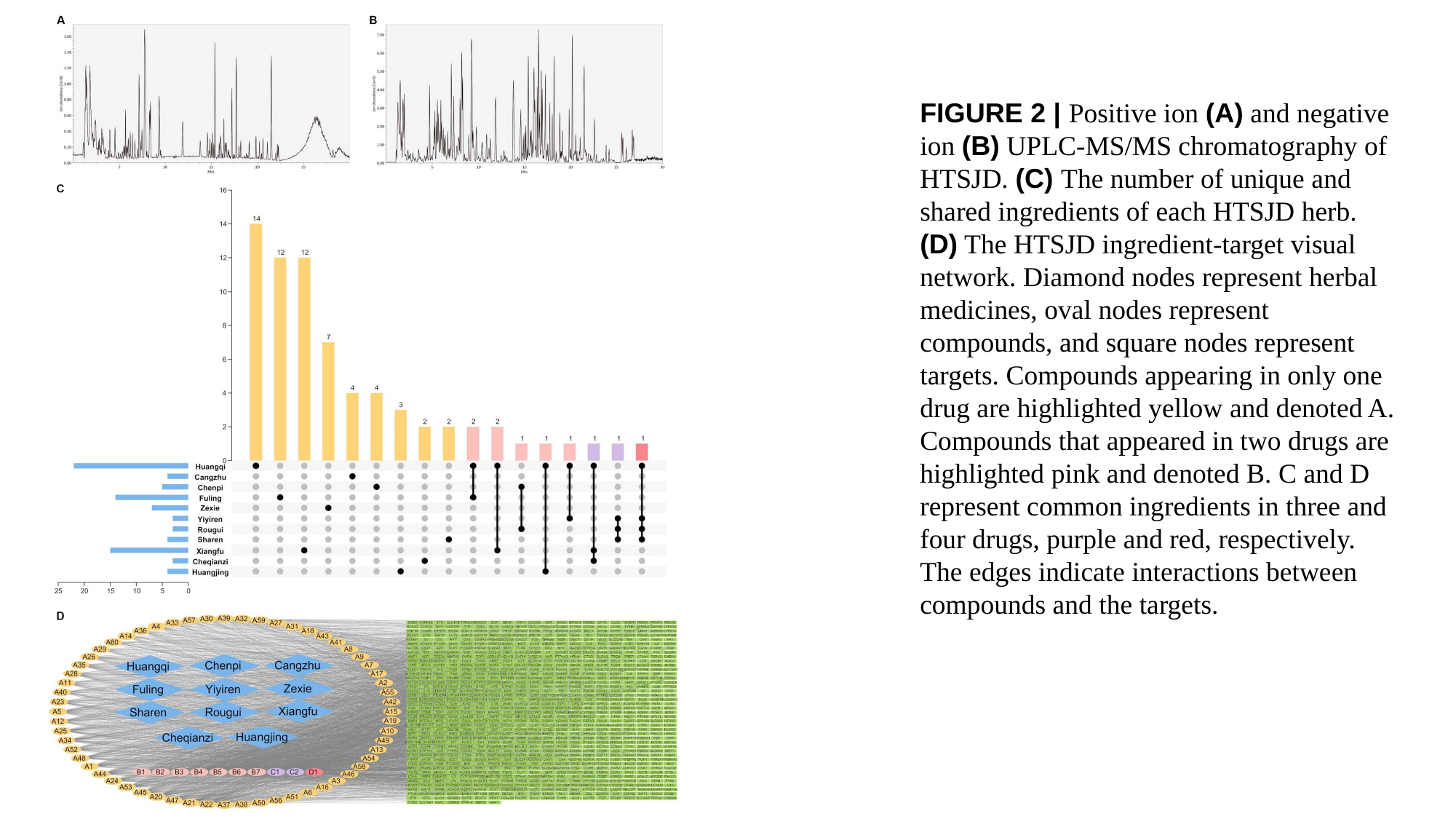

FIGURE 2 | Positive ion (A) and negative ion (B) UPLC-MS/MS chromatography of HTSJD. (C) The number of unique and shared ingredients of each HTSJD herb. (D) The HTSJD ingredient-target visual network. Diamond nodes represent herbal medicines, oval nodes represent compounds, and square nodes represent targets. Compounds appearing in only one drug are highlighted yellow and denoted A. Compounds that appeared in two drugs are highlighted pink and denoted B. C and D represent common ingredients in three and four drugs, purple and red, respectively. The edges indicate interactions between compounds and the targets.

## Slide 3
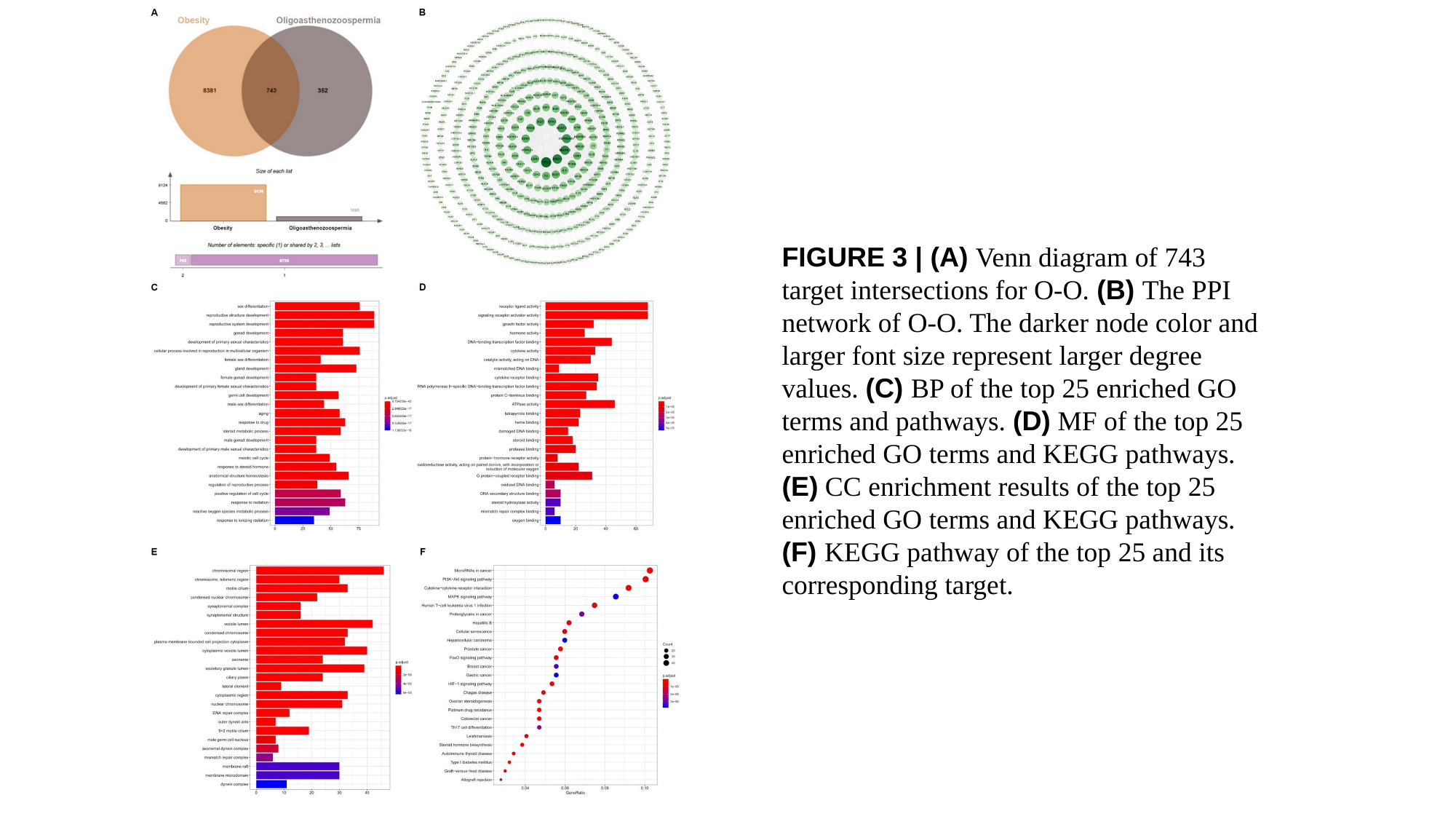

FIGURE 3 | (A) Venn diagram of 743 target intersections for O-O. (B) The PPI network of O-O. The darker node color and larger font size represent larger degree values. (C) BP of the top 25 enriched GO terms and pathways. (D) MF of the top 25 enriched GO terms and KEGG pathways. (E) CC enrichment results of the top 25 enriched GO terms and KEGG pathways. (F) KEGG pathway of the top 25 and its corresponding target.

## Slide 4
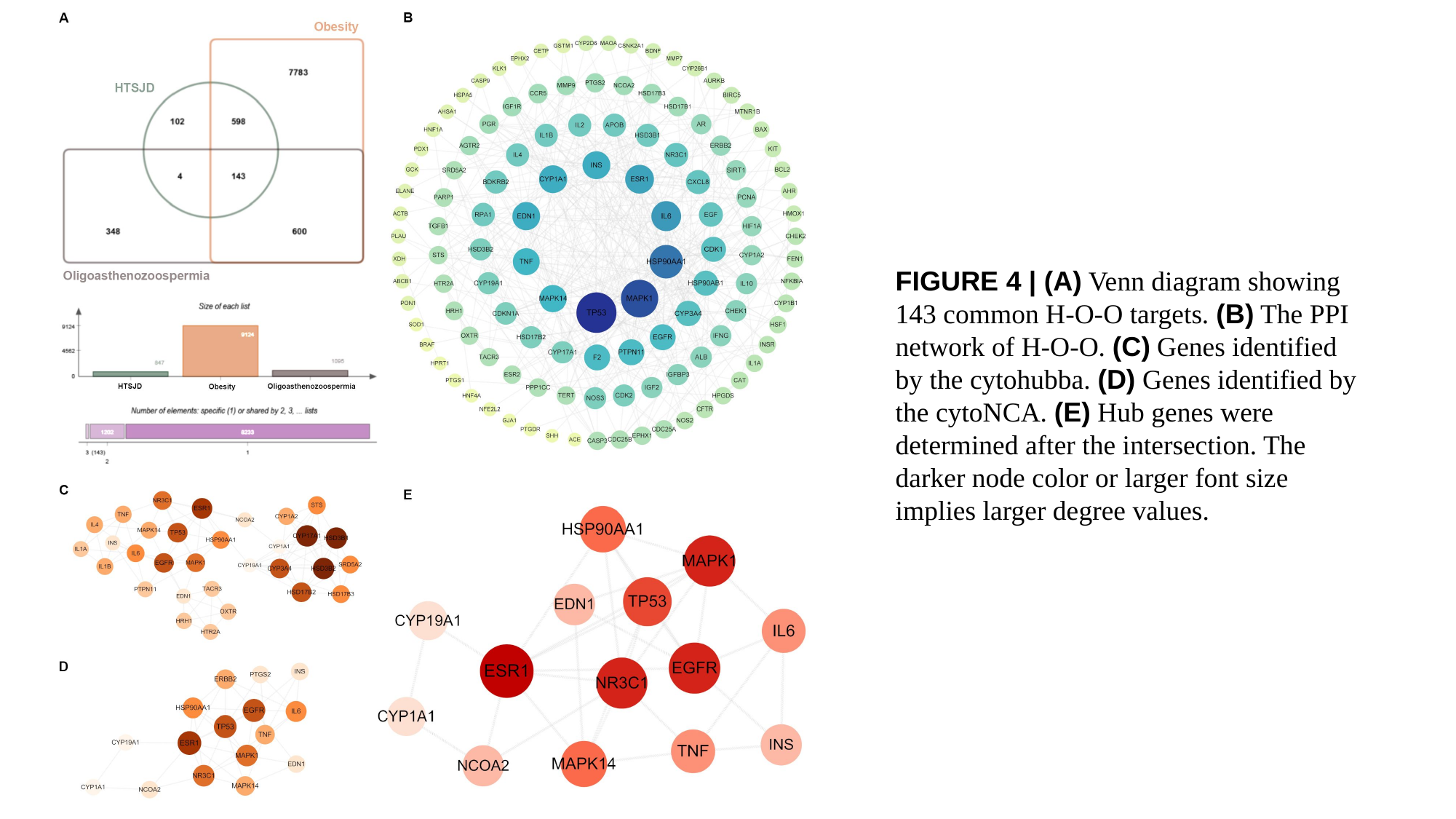

FIGURE 4 | (A) Venn diagram showing 143 common H-O-O targets. (B) The PPI network of H-O-O. (C) Genes identified by the cytohubba. (D) Genes identified by the cytoNCA. (E) Hub genes were determined after the intersection. The darker node color or larger font size implies larger degree values.

## Slide 5
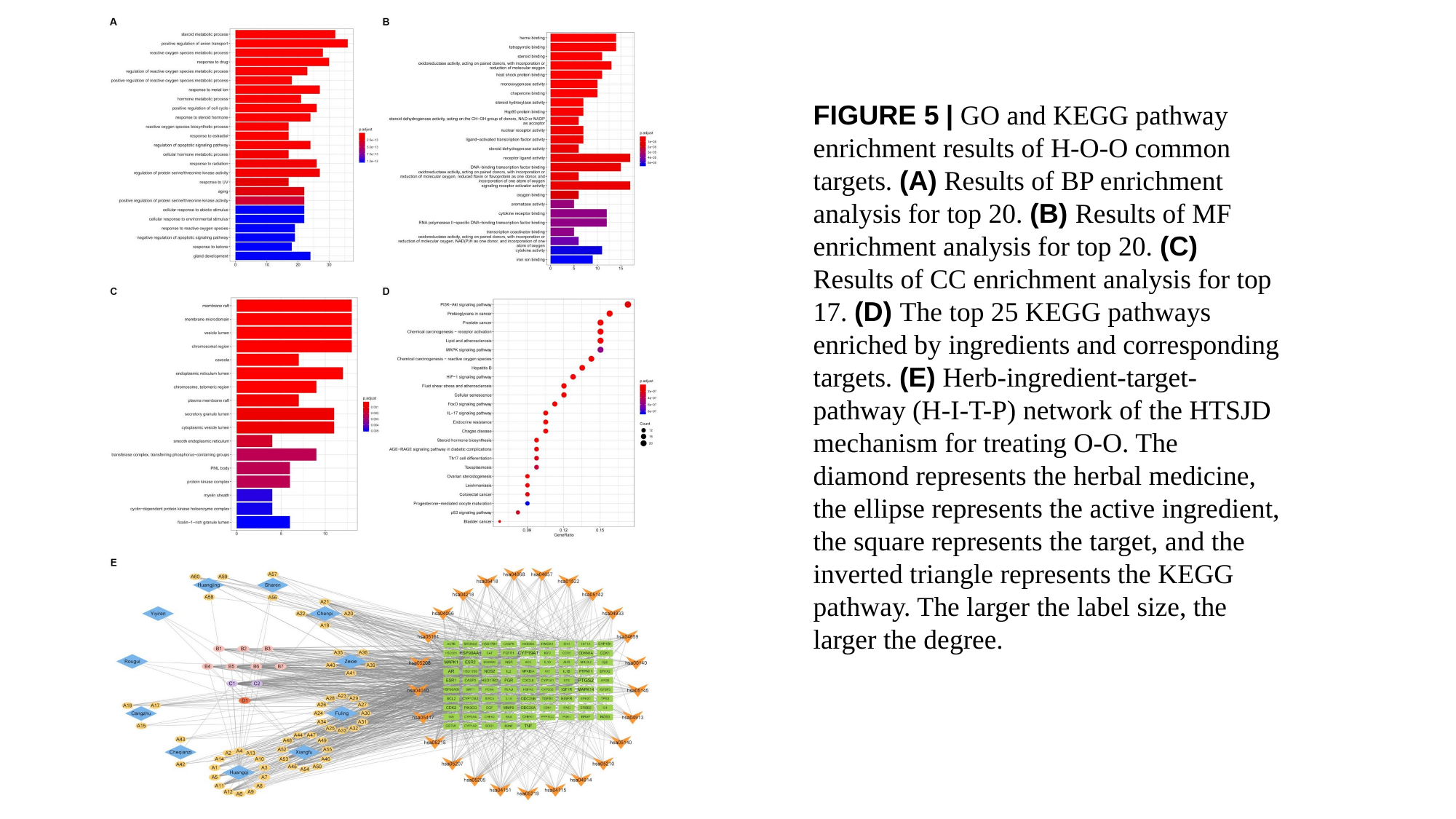

FIGURE 5 | GO and KEGG pathway enrichment results of H-O-O common targets. (A) Results of BP enrichment analysis for top 20. (B) Results of MF enrichment analysis for top 20. (C) Results of CC enrichment analysis for top 17. (D) The top 25 KEGG pathways enriched by ingredients and corresponding targets. (E) Herb-ingredient-target-pathway (H-I-T-P) network of the HTSJD mechanism for treating O-O. The diamond represents the herbal medicine, the ellipse represents the active ingredient, the square represents the target, and the inverted triangle represents the KEGG pathway. The larger the label size, the larger the degree.

## Slide 6
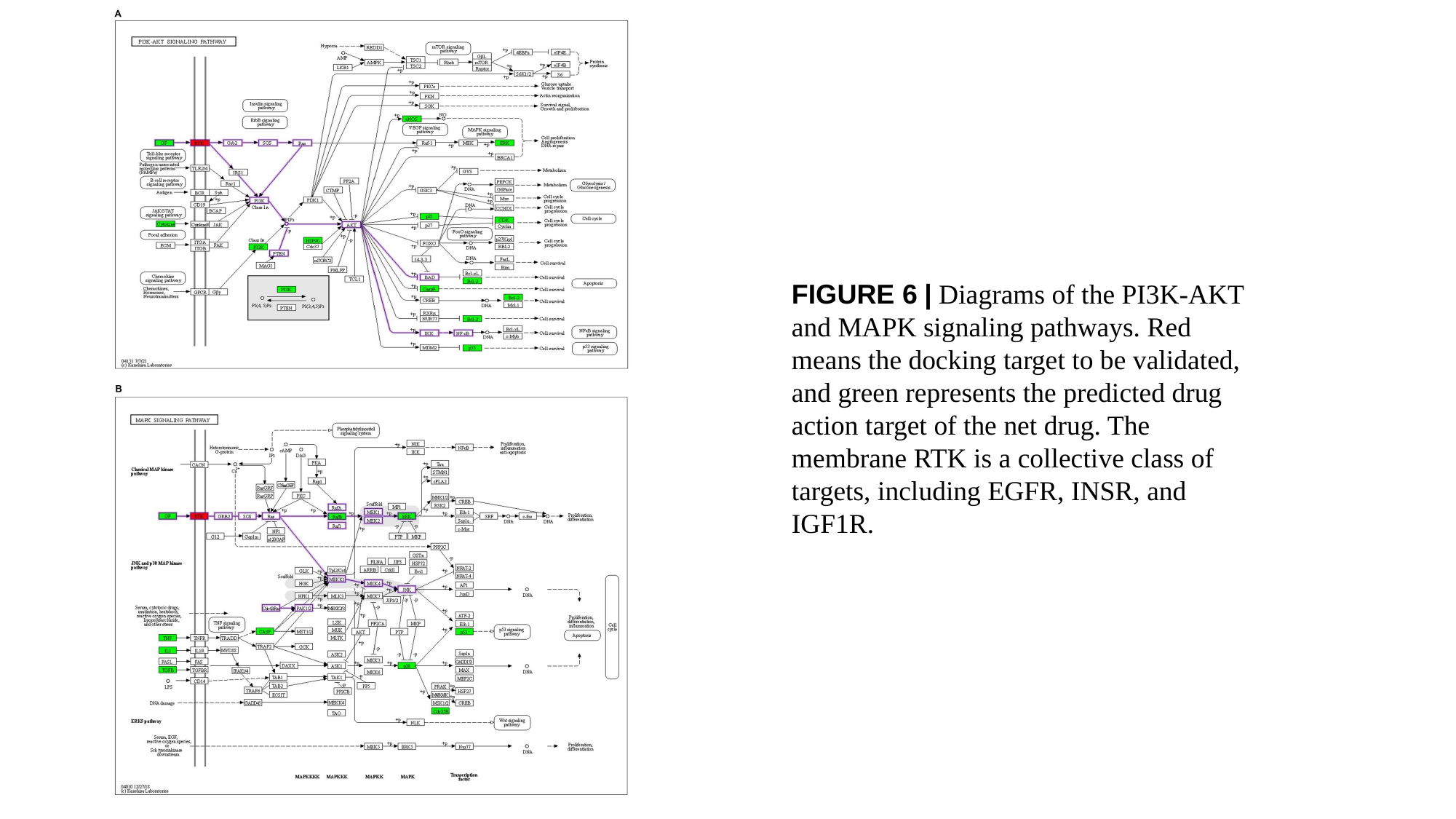

FIGURE 6 | Diagrams of the PI3K-AKT and MAPK signaling pathways. Red means the docking target to be validated, and green represents the predicted drug action target of the net drug. The membrane RTK is a collective class of targets, including EGFR, INSR, and IGF1R.

## Slide 7
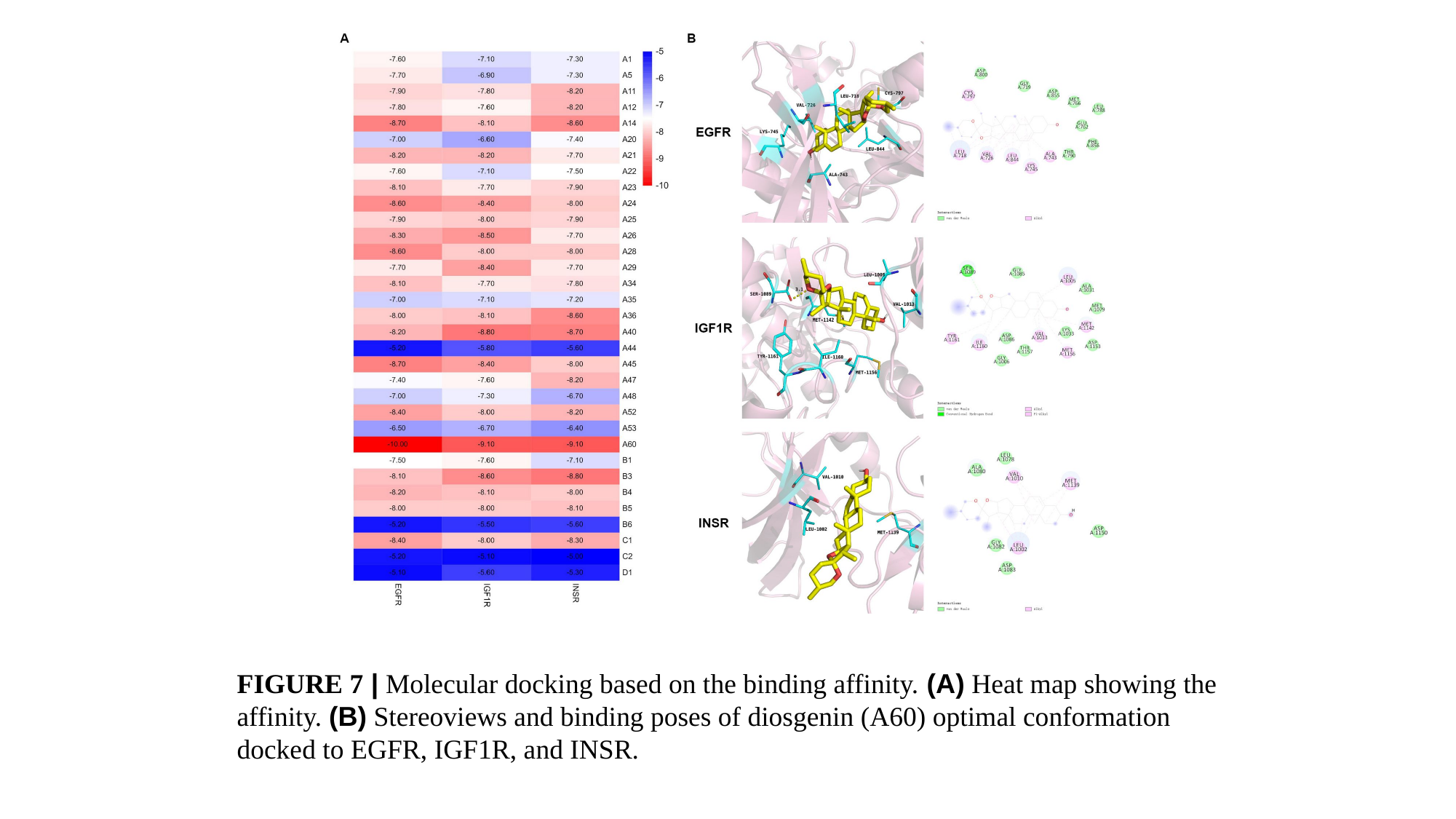

FIGURE 7 | Molecular docking based on the binding affinity. (A) Heat map showing the affinity. (B) Stereoviews and binding poses of diosgenin (A60) optimal conformation docked to EGFR, IGF1R, and INSR.

## Slide 8
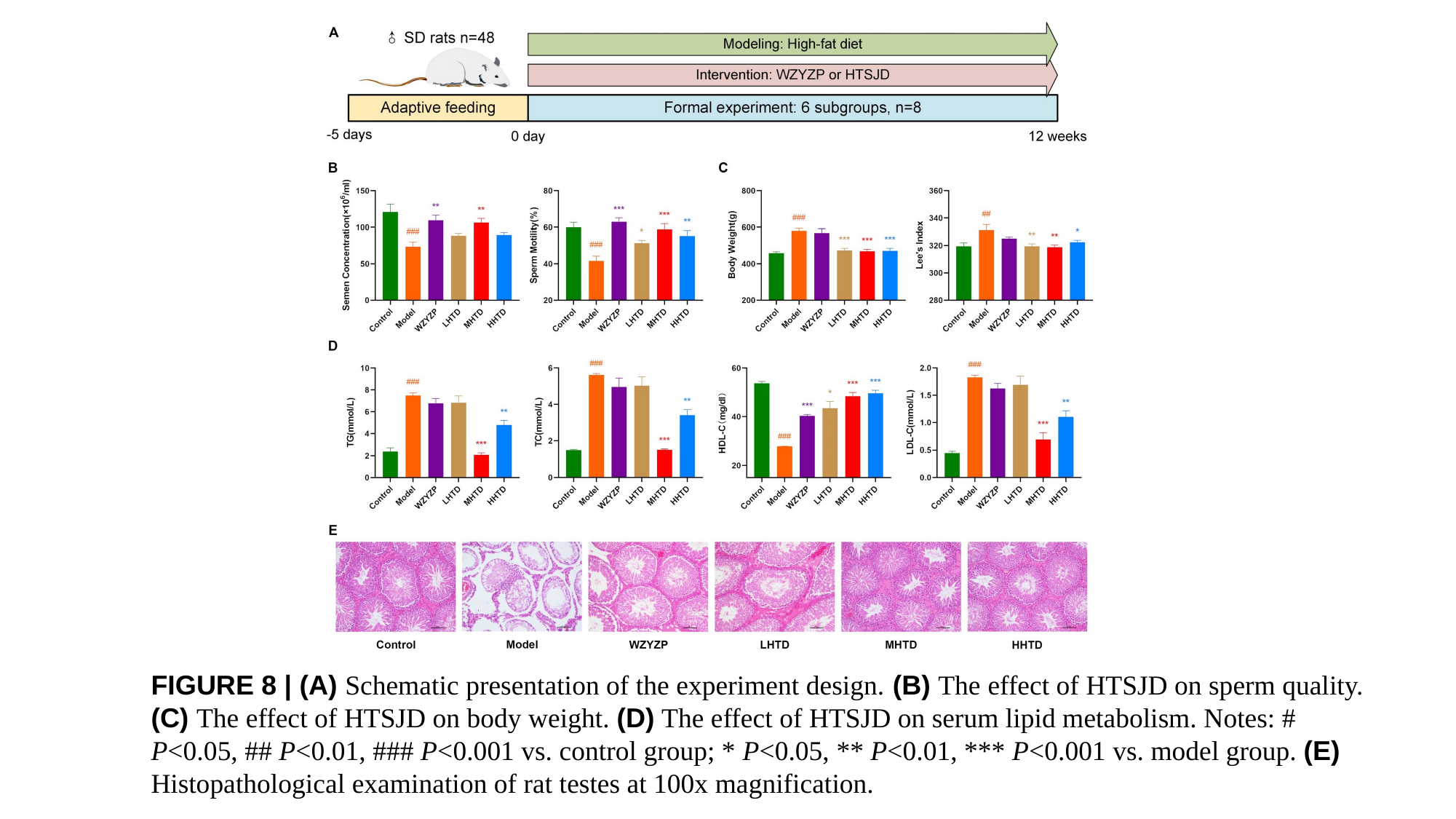

FIGURE 8 | (A) Schematic presentation of the experiment design. (B) The effect of HTSJD on sperm quality. (C) The effect of HTSJD on body weight. (D) The effect of HTSJD on serum lipid metabolism. Notes: # P<0.05, ## P<0.01, ### P<0.001 vs. control group; * P<0.05, ** P<0.01, *** P<0.001 vs. model group. (E) Histopathological examination of rat testes at 100x magnification.

## Slide 9
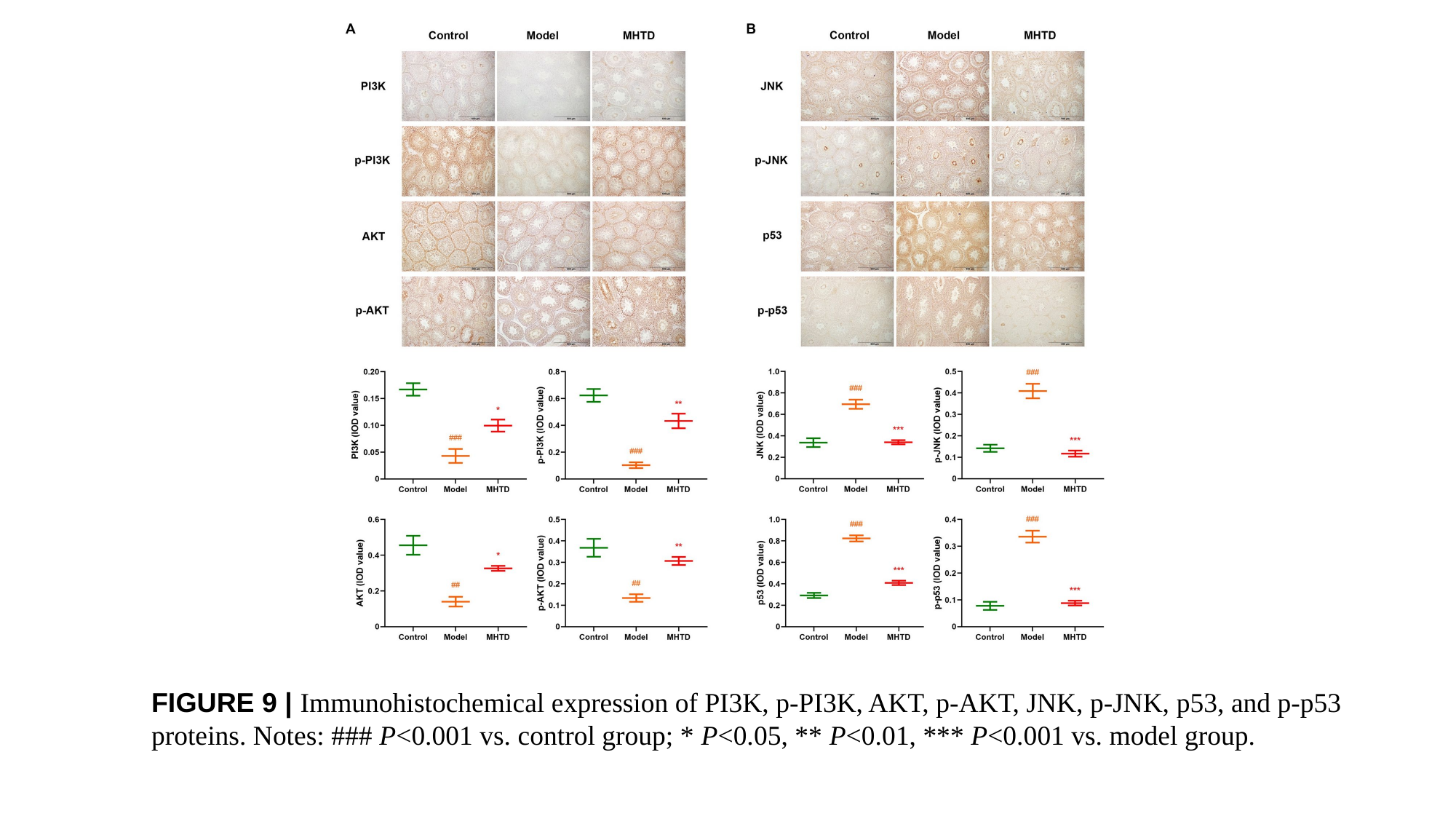

FIGURE 9 | Immunohistochemical expression of PI3K, p-PI3K, AKT, p-AKT, JNK, p-JNK, p53, and p-p53 proteins. Notes: ### P<0.001 vs. control group; * P<0.05, ** P<0.01, *** P<0.001 vs. model group.
