# Supplementary material for: Hua-Tan-Sheng-Jing Decoction Treats Obesity With Oligoasthenozoospermia by Up-Regulating the PI3K-AKT and Down-Regulating the JNK MAPK Signaling Pathways: At the Crossroad of Obesity and Oligoasthenozoospermia
Source: Front Pharmacol. 2022 Apr 26;13:896434. doi: 10.3389/fphar.2022.896434 (PMC9086321; doi:10.3389/fphar.2022.896434)
Supplement: Supplementary file 2 [file DataSheet1.docx]

**Table S1**

The herb information of HTSJD and traditional uses of each herb in the Chinese pharmacopeia (2020 edition)

| **Medicine** | | **Medicinal parts** | **Batch number** | **Traditional and clinical uses** |
| --- | --- | --- | --- | --- |
| **English name** | **Chinese name** |  |  |  |
| Astragali Radix | Huangqi | Dried root | 17022602 | Qi deficiency and weakness, lack of food and loose stool, depression of middle qi, prolapse of anus for a long time, blood collapse and leakage, surface deficiency from sweat, qi deficiency edema, internal heat and thirst, blood deficiency withering yellow, hemiplegia, arthralgia numbness, carbuncle difficult to collapse, long collapse not convergent. |
| Atractylodis Rhizoma | Cangzhu | Dried rhizome | 17002791 | Dampness blocking middle coke, abdominal distension, diarrhea, edema, beriberi weakness, rheumatism arthralgia, wind cold, night blindness, dizziness |
| Citri Reticulatae Pericarpium | Chenpi | Dry ripe pericarp | 17023892 | Abdominal distension, less food, vomiting and diarrhea, coughing more phlegm |
| Poria | Fuling | Dry sclerotia | 17024072 | Edema, less urine, phlegm, dizziness, palpitations, spleen deficiency, lack of food, loose stool, restlessness, palpitations and insomnia |
| Alismatis Rhizoma | Zexie | Dried tuber | 17022012 | Adverse urination, edema and fullness, less diarrhea, dizziness of phlegm, astringent pain of hot rain, hyperlipidemia |
| Coicis Semen | Yiyiren | Mature kernel | 17018562 | Edema, beriberi, adverse urination, spleen deficiency diarrhea, damp arthralgia contracture, pulmonary carbuncle, intestinal carbuncle, warts, cancer |
| Cinnamomi Cortex | Rougui | Dry bark | 17018491 | Impotence，uterine cold, waist and knee cold pain, kidney deficiency for asthma, deficiency yang floating, dizziness, heart and abdomen cold pain, deficiency cold vomiting and diarrhea, cold hernia and abdominal pain, dysmenorrhea |
| Amomi Fructus | Sharen | Dried ripe fruit | 17001113 | Dampness and turbid obstruction, epigastric ruffian not hungry, spleen and stomach deficiency and cold, vomiting and diarrhea, malignant obstruction of pregnancy, restless fetal movement |
| Cyperi Rhizoma | Xiangfu | Dried rhizome | 17018784 | Qi stagnation, chest pain, hernia pain, breast distension pain, spleen and stomach qi stagnation, epigastric distension, fullness pain, irregular menstruation, amenorrhea and dysmenorrhea |
| Plantaginis Semen | Cheqianzi | Dry mature seed | 17024016 | Heat strangury and astringent pain, edema and fullness, summer heat and dampness diarrhea, red swelling and pain in the eyes, phlegm-heat cough |
| Polygonati Rhizoma | Huangjing | Dried rhizome | 17024031 | Spleen and stomach qi deficiency, body fatigue, stomach yin deficiency, dry mouth and less food, lung deficiency and dryness cough, labor cough and hemoptysis, lack of essence blood, sour and soft waist and knees, early whitening, internal heat to eliminate thirst |

**Table S2**

Positive ion chromatography (TIC) of HTSJD based on UPLC-MS/MS.

| **PeakID** | **Title** | **RT (min)** | **Area** | **Adduct** | **Total score** |
| --- | --- | --- | --- | --- | --- |
| 4 | Dihydrocapsaicin | 1.007033 | 31248.47 | [M+H]+ | 81.2 |
| 197 | allantoin | 1.103117 | 35991.92 | [M+H]+ | 81.4 |
| 263 | vanillic acid | 1.110283 | 60775.77 | [M+H]+ | 80.4 |
| 303 | benzoic acid | 1.118117 | 106705.4 | [M+H]+ | 80.7 |
| 711 | nicotinic acid | 1.249117 | 9593.278 | [M+H]+ | 81.2 |
| 714 | FERULIC ACID (CIS) | 1.249117 | 4879.443 | [M+H]+ | 81.6 |
| 784 | proanthocyanidin b2 | 1.257617 | 3770.688 | [M+H]+ | 80.2 |
| 814 | Geniposidic acid | 1.264783 | 18831.23 | [M+H]+ | 80.5 |
| 946 | papaverine | 1.28745 | 129123.5 | [M+H]+ | 81.3 |
| 962 | choline | 1.294283 | 139232 | [M+H]+ | 80.8 |
| 978 | l-alpha-alanine | 1.301783 | 28517.96 | [M+H]+ | 80.9 |
| 979 | homoserine | 1.301783 | 285390.7 | [M+H]+ | 81.3 |
| 993 | azetidine-2-carboxylic acid | 1.308617 | 629863.3 | [M+H]+ | 80.6 |
| 995 | cinnamaldehyde | 1.308617 | 189235.7 | [M+H]+ | 80.9 |
| 999 | prunasin | 1.308617 | 66162.32 | [M+H]+ | 80.5 |
| 1071 | 9,10-dimethoxy-pterocarpane-3-o-beta-d-glucoside | 1.323117 | 23094.48 | [M+H]+ | 81.7 |
| 1144 | 2-Pyrol | 1.344767 | 64142.32 | [M+H]+ | 81.2 |
| 1167 | aspartic acid | 1.3516 | 14186.46 | [M+H]+ | 81 |
| 1264 | HMF | 1.373267 | 190837.8 | [M+H]+ | 81.5 |
| 1266 | Dimethyl L-malate | 1.373267 | 512000.6 | [M+H]+ | 81.1 |
| 1306 | 2-lauroleic acid | 1.3801 | 14759.67 | [M+H]+ | 81.7 |
| 1322 | alpha-D-Galp-(1->6)-alpha-D-Galp-(1->6)-D-Glu | 1.3801 | 120463.3 | [M+H]+ | 81.7 |
| 1336 | naphthalene | 1.387433 | 140231.2 | [M+H]+ | 80.1 |
| 1339 | quinic acid | 1.387433 | 46668.04 | [M+H]+ | 81.3 |
| 1463 | Atractyloyne | 1.451433 | 21329.12 | [M+H]+ | 81 |
| 1520 | verbascose | 1.4731 | 125104 | [M+H]+ | 81.5 |
| 1607 | Beta-Glucan | 1.501933 | 33322.53 | [M+H]+ | 81.3 |
| 1626 | cinnamicalcohol | 1.516433 | 22508.76 | [M+H]+ | 81.2 |
| 1629 | cinnamyl acetate | 1.516433 | 3282952 | [M+H]+ | 81.3 |
| 1725 | Fucopyranose, L- | 1.5976 | 74274.63 | [M+H]+ | 80.5 |
| 1732 | sucrose | 1.5976 | 894543.7 | [M+H]+ | 80.6 |
| 1791 | Dimethyl anthranilate | 1.6596 | 63142.99 | [M+H]+ | 81.5 |
| 1831 | alpha-D-fructofuranose | 1.6756 | 430546.5 | [M+H]+ | 81.8 |
| 1962 | 2-deoxy-d-ribono-1,4-lactone | 1.792083 | 26259.54 | [M+H]+ | 81.1 |
| 2156 | (2E,8E)-9-(2-furyl)nona-2,8-dien-4,6-diyn-1-ol | 1.932917 | 33732.93 | [M+H]+ | 81.2 |
| 2171 | codeine | 1.947083 | 28782.84 | [M+H]+ | 82.3 |
| 2200 | 2,3-Dihydro-4-hydroxy-2-indole-3-acetonitrile | 1.968417 | 166157.8 | [M+H]+ | 82 |
| 2257 | Nonox D | 2.03475 | 50364.87 | [M+H]+ | 80.4 |
| 2280 | adenine | 2.064583 | 1719029 | [M+H]+ | 80.6 |
| 2281 | gamma-aminobutyric acid | 2.071567 | 78607.58 | [M+H]+ | 81.2 |
| 2373 | l-leucine | 2.145067 | 69043.08 | [M+H]+ | 82.1 |
| 2396 | catechol | 2.1599 | 60513.34 | [M+H]+ | 82.4 |
| 2429 | 3,5-dimethoxystilbene | 2.182567 | 28700.84 | [M+H]+ | 80.1 |
| 2533 | coixol | 2.2719 | 29061.9 | [M+H]+ | 82.1 |
| 2571 | octopamine | 2.2934 | 13821.03 | [M+H]+ | 82.4 |
| 2719 | synepherine | 2.400717 | 829016.7 | [M+H]+ | 82.6 |
| 2747 | BZM | 2.414883 | 54931.63 | [M+H]+ | 81.6 |
| 2828 | AKG | 2.547883 | 130922.1 | [M+H]+ | 80.8 |
| 2885 | Prolinum | 2.57605 | 60839.71 | [M+H]+ | 82.6 |
| 2965 | Vanay | 2.664217 | 18464.52 | [M+H]+ | 83 |
| 3037 | 3-Hydroxy-2-picoline | 2.709717 | 100827.8 | [M+H]+ | 81.2 |
| 3040 | 3β-hydroxyatractylone | 2.709717 | 14343.92 | [M+H]+ | 82.6 |
| 3394 | n-butyl-β-D-fructopyranoside | 2.7952 | 24622.67 | [M+H]+ | 80.3 |
| 3493 | 3β-acetoxyatractylone | 2.819367 | 22733.61 | [M+H]+ | 82.5 |
| 3521 | 3β-hydroxylanosta-7,9(11),24-trien-21-oic acid | 2.826867 | 76692.3 | [M+H]+ | 82.7 |
| 3603 | α-hederin | 2.8662 | 24360.98 | [M+H]+ | 83 |
| 3658 | 16β-hydroxyalisol B monoacetate | 2.881533 | 47319.11 | [M+H]+ | 83.2 |
| 3706 | (?)-norrotundene | 2.9437 | 59617.59 | [M+H]+ | 83.1 |
| 3724 | β-amyrin acetate | 2.966867 | 52240.75 | [M+2H]2+ | 81.5 |
| 3779 | 13β,17β-epoxyalisol a | 2.999367 | 187317 | [M+H]+ | 82.3 |
| 3835 | 3β-p-hydroxybenzoyldehydrotumulosicacid | 3.015533 | 71362.73 | [M+H]+ | 81.3 |
| 3874 | 3β-hydroxy-16α-acetoxy-lanosta-7,9(11),24-trien-21-oicacid | 3.0242 | 13035.79 | [M+H]+ | 81 |
| 3974 | 13β,17β-epoxyalisol a 24-acetate | 3.048033 | 21059.24 | [M+H]+ | 83 |
| 4050 | 16β-methoxyalisol B monoacetate | 3.056533 | 37807.27 | [M+H]+ | 80.9 |
| 4083 | elemicin | 3.0642 | 157250.1 | [M+H]+ | 82.1 |
| 4132 | adeninenucleoside | 3.088867 | 1395878 | [M+H]+ | 83.1 |
| 4240 | Proantho Cyanidins | 3.12485 | 7285.76 | [M+H]+ | 81.7 |
| 4244 | beta-elemene | 3.13235 | 44068.3 | [M+H]+ | 83.2 |
| 4305 | cinnamyl benzoate | 3.154017 | 126452 | [M+H]+ | 80.2 |
| 4335 | Sulfoorientalol C | 3.16185 | 123881.4 | [M+H]+ | 81.4 |
| 4350 | paeonol | 3.169017 | 10855.3 | [M+H]+ | 82 |
| 4362 | (Z)-1-(2,4-dihydroxyphenyl)-3-(4-hydroxyphenyl)prop-2-en-1-one | 3.177017 | 24791.75 | [M+H]+ | 80.5 |
| 4377 | MLT | 3.184517 | 9956.593 | [M+H]+ | 83.3 |
| 4380 | n-methyltyramine | 3.184517 | 8384.902 | [M+H]+ | 82.1 |
| 4383 | guanosine | 3.184517 | 581070 | [M+H]+ | 82.5 |
| 4396 | 2H-pyran | 3.19285 | 4399.681 | [M+H]+ | 81.8 |
| 4554 | nomilin | 3.30335 | 40417.37 | [M+H]+ | 81.6 |
| 4650 | 4,7-dimethyl-1-tetralone | 3.400683 | 81820.89 | [M+H]+ | 81.5 |
| 4719 | Solavetivone | 3.4575 | 23612.06 | [M+H]+ | 82.6 |
| 4735 | Ethyl glucoside | 3.482333 | 31029.98 | [M+H]+ | 80.2 |
| 4755 | catalpol | 3.489833 | 132776.5 | [M+H]+ | 83.3 |
| 4800 | NO-biletin | 3.548667 | 35956.13 | [M+H]+ | 82.1 |
| 4809 | 7-O-methylisomucronulatol | 3.565333 | 55482.79 | [M+H]+ | 82.6 |
| 4839 | aucubigenin | 3.6155 | 20879.34 | [M+H]+ | 83.7 |
| 4903 | caffeic acid dimethyl ether | 3.698667 | 10486.58 | [M+H]+ | 84 |
| 4962 | acetyl atractylodinol | 3.756333 | 27896.74 | [M+H]+ | 82.7 |
| 5080 | PHB | 3.881983 | 16214.06 | [M+H]+ | 84.1 |
| 5139 | 2-Acridinecarboxylic acid | 3.969817 | 31516.27 | [M+H]+ | 82 |
| 5151 | canavanine | 3.977483 | 13198.93 | [M+H]+ | 82.5 |
| 5241 | atractylon | 4.109317 | 43011.86 | [M+H]+ | 83.5 |
| 5332 | protocatechuic acid | 4.2743 | 13333.73 | [M+H]+ | 84.6 |
| 5359 | L-Milchsaeure | 4.323967 | 8273.452 | [M+H]+ | 82.3 |
| 5368 | tetramethylpyrazine | 4.340633 | 15101.32 | [M+H]+ | 84.6 |
| 5397 | alpha-Thymidine | 4.382633 | 29701.52 | [M+H]+ | 84.4 |
| 5418 | Flavaxin | 4.423467 | 42192.29 | [M+H]+ | 83.1 |
| 5482 | cinnamic acid | 4.530117 | 72413.64 | [M+H]+ | 83.2 |
| 5514 | atractulodin | 4.559783 | 18658.95 | [M+H]+ | 83.3 |
| 5564 | (2S)-4-methoxy-7-methyl-2-[1-methyl-1-[(2S,3R,4S,5S,6R)-3,4,5-trihydroxy-6-methylol-tetrahydropyran-2-yl]oxy-ethyl]-2,3-dihydrofuro[3,2-g]chromen-5-one | 4.59095 | 34612.34 | [M+H]+ | 80.3 |
| 5585 | isoflavanone | 4.63095 | 44669.86 | [M+H]+ | 80.2 |
| 5628 | (+)-eudesma-4(15),7(11)-dien-8-one | 4.697117 | 7687.914 | [M+H]+ | 81.8 |
| 5634 | aucubin | 4.70595 | 12192.82 | [M+H]+ | 85 |
| 5687 | n-candicine | 4.796283 | 13369.88 | [M+H]+ | 85.1 |
| 5735 | colchamine | 4.862617 | 38028.13 | [M+H]+ | 82.4 |
| 5892 | 2-hydroxy-4-methyl acetophenone | 5.028767 | 22949.93 | [M+H]+ | 84.9 |
| 5905 | coixan A | 5.037766 | 21010.12 | [M+H]+ | 83.1 |
| 5961 | ethyl gallate | 5.1121 | 19129.78 | [M+H]+ | 82.6 |
| 5990 | 10-epiatractyloside a | 5.137933 | 16743.63 | [M+H]+ | 81.4 |
| 6040 | ellipticine | 5.204767 | 29531.47 | [M+H]+ | 80.8 |
| 6078 | Heriguard | 5.26225 | 48041.56 | [M+H]+ | 85 |
| 6097 | porphyroxine | 5.287917 | 95334.85 | [M+H]+ | 85.1 |
| 6098 | syringin | 5.287917 | 39597.89 | [M+H]+ | 85.1 |
| 6102 | styrene | 5.295583 | 8697.507 | [M+H]+ | 84.5 |
| 6113 | coumarin | 5.312417 | 17284.7 | [M+H]+ | 85.4 |
| 6147 | formononetin | 5.345917 | 21334.48 | [M+H]+ | 80.8 |
| 6171 | 2-furancarboxylicacid | 5.354917 | 5124.046 | [M+H]+ | 80.8 |
| 6196 | cryptopine | 5.379583 | 50025.38 | [M+H]+ | 80.2 |
| 6261 | Cleomin | 5.489083 | 58538.32 | [M+H]+ | 85.8 |
| 6269 | tryptophan | 5.489083 | 647892.3 | [M+H]+ | 85.6 |
| 6287 | Flavonol | 5.50575 | 5067.5 | [M+H]+ | 81.6 |
| 6337 | n-butyl-β-D-fructopyranoside | 5.563583 | 24624.41 | [M+H]+ | 85.3 |
| 6353 | sedoheptulose | 5.589417 | 7556.317 | [M+H]+ | 84.8 |
| 6368 | thebaine | 5.6064 | 84157.91 | [M+H]+ | 82.6 |
| 6399 | myristicin | 5.6479 | 58696.87 | [M+H]+ | 85.1 |
| 6416 | rutin | 5.657067 | 118898 | [M+H]+ | 85.7 |
| 6418 | suffruticoside a | 5.657067 | 10576.13 | [M+H]+ | 82.2 |
| 6501 | anthracene | 5.754567 | 9587.373 | [M+H]+ | 84.7 |
| 6537 | Ammiol | 5.7964 | 7253.019 | [M+H]+ | 84.4 |
| 6565 | inositol | 5.830067 | 8160.681 | [M+H]+ | 83.7 |
| 6625 | icariside f2 | 5.880733 | 141067.6 | [M+H]+ | 85.8 |
| 6659 | Sennoside C | 5.9144 | 6803.77 | [M+H]+ | 86.2 |
| 6690 | nonyl vanillylamide | 5.957383 | 27537.89 | [M+H]+ | 83.1 |
| 6705 | 5,4'-dihydroxyflavone-6-c-β-d-glycosyl-rhamnoside-7-o-glycoside | 5.96505 | 53926.26 | [M+H]+ | 84.9 |
| 6749 | anisaldehyde | 5.998883 | 18037.13 | [M+H]+ | 86.1 |
| 6780 | 3'-Hydroxy-4'-methoxyisoflavone-7-O-beta-D-glucoside | 6.007884 | 57604.18 | [M+H]+ | 86 |
| 6804 | atsudaidai | 6.049217 | 36273.26 | [M+H]+ | 86.1 |
| 6810 | naringenin | 6.058717 | 28022.67 | [M+H]+ | 86.3 |
| 6818 | naringin | 6.058717 | 210587.3 | [M+H]+ | 86 |
| 6898 | 4-hydroxy-3,5-dimethoxyacetophenone | 6.13505 | 15686.39 | [M+H]+ | 84.1 |
| 6912 | catechin | 6.144217 | 15898 | [M+H]+ | 84 |
| 6962 | beta-citraurin | 6.186383 | 40721.65 | [M+H]+ | 86.5 |
| 6974 | 3β-acetoxyatractylone | 6.19555 | 143005 | [M+H]+ | 85.5 |
| 6992 | p-cymene | 6.211717 | 19606.15 | [M+H]+ | 86.5 |
| 7013 | anethole | 6.228717 | 10172.57 | [M+H]+ | 84.8 |
| 7081 | Trimethyl citrate | 6.263216 | 21899.5 | [M+H]+ | 83.3 |
| 7086 | Plantaginin | 6.263216 | 10846.26 | [M+H]+ | 86.5 |
| 7089 | rhamnocitrin-3-O-glucoside | 6.270884 | 24629.79 | [M+H]+ | 85.1 |
| 7098 | rhamnocitrin | 6.280217 | 24787.95 | [M+H]+ | 86.4 |
| 7111 | atractyloside d | 6.2882 | 17191.79 | [M+H]+ | 80.4 |
| 7131 | cis-isoascaridole | 6.322866 | 18014.77 | [M+H]+ | 86.4 |
| 7165 | vitexin xyloside | 6.3572 | 14599.74 | [M+H]+ | 83.2 |
| 7230 | vanillin | 6.425367 | 6016.348 | [M+H]+ | 86.4 |
| 7250 | orientalol e | 6.441867 | 7623.103 | [M+H]+ | 84.1 |
| 7251 | capsaicin | 6.441867 | 20176.64 | [M+H]+ | 86.6 |
| 7258 | albiflorin | 6.4507 | 98119.05 | [M+H]+ | 86.7 |
| 7282 | 2'-O-beta-Glucopyranosyl-5,7,4',5'-tetrahydroxyflavanone | 6.484867 | 21682.42 | [M+H]+ | 80.1 |
| 7296 | citral | 6.502367 | 26308.54 | [M+H]+ | 86.2 |
| 7307 | atractyloside i | 6.5102 | 85794.05 | [M+H]+ | 86.7 |
| 7319 | Lariciresinol | 6.519533 | 19935.89 | [M+H]+ | 80.3 |
| 7345 | (2S)-7-[(2S,3R,4S,5S,6R)-4,5-dihydroxy-6-methylol-3-[(2S,3R,4R,5R,6S)-3,4,5-trihydroxy-6-methyl-tetrahydropyran-2-yl]oxy-tetrahydropyran-2-yl]oxy-5-hydroxy-2-(3-hydroxy-5-methoxy-phenyl)chroman-4-one | 6.544367 | 84338.76 | [M+H]+ | 86.8 |
| 7401 | anemonin | 6.588533 | 94852.05 | [M+H]+ | 86.7 |
| 7455 | galloylpaeoniflorin | 6.675517 | 10655.64 | [M+H]+ | 86.2 |
| 7481 | 3β-hydroxylanosta-7,9(11),24-trien-21-oic acid | 6.69235 | 27065.09 | [M+H]+ | 83.2 |
| 7534 | paeoniflorgenone | 6.749683 | 10781.2 | [M+H]+ | 86.8 |
| 7549 | (6aR,11aR)-9,10-dimethoxy-6a,11a-dihydro-6H-benzofurano[3,2-c]chromen-3-ol | 6.759517 | 45451.57 | [M+H]+ | 86.8 |
| 7655 | Hirsutrin | 6.881017 | 22719.88 | [M+H]+ | 85 |
| 7667 | quercetin | 6.88885 | 17116.81 | [M+H]+ | 87.2 |
| 7881 | calycosin | 7.142833 | 347440.7 | [M+H]+ | 87.2 |
| 7974 | (+)-Catechin-5-O-glucoside | 7.213 | 110563.8 | [M+H]+ | 87 |
| 8003 | DIBP | 7.272 | 38572.45 | [M+H]+ | 87.5 |
| 8004 | 1,7-Dihydroxy-3,9-dimethoxy pterocarpene | 7.272 | 11988.95 | [M+H]+ | 87.5 |
| 8011 | 7,2'-dihydroxy-3',4'-dimethoxyisoflavone-7-O-β-D-glucoside | 7.279667 | 86641.55 | [M+H]+ | 87.3 |
| 8035 | Citromitin | 7.3145 | 20022.9 | [M+H]+ | 84.2 |
| 8036 | khellol glucoside | 7.3145 | 37503.09 | [M+H]+ | 84.5 |
| 8071 | p-hydroxybenzyl alcohol | 7.347667 | 5644.338 | [M+H]+ | 87.6 |
| 8111 | Sulfoorientalol A | 7.371483 | 15041.8 | [M+H]+ | 86.8 |
| 8151 | Agrimol C | 7.402667 | 270313.5 | [M+H]+ | 82.2 |
| 8171 | Polystachoside | 7.413983 | 20301.56 | [M+H]+ | 87.6 |
| 8176 | hesperidin methyl chalcone | 7.413983 | 32190.9 | [M+H]+ | 87.6 |
| 8182 | Calceolarioside A | 7.422317 | 21231.84 | [M+H]+ | 87.6 |
| 8228 | o-acetylpachymicacid-25-ol | 7.459317 | 646365.3 | [M+H]+ | 84.4 |
| 8235 | tangeretin | 7.466983 | 38066.28 | [M+H]+ | 87.6 |
| 8262 | luteolin-7-beta-d-glucuronide | 7.502316 | 8509.432 | [M+H]+ | 87.6 |
| 8263 | clemaphenolA | 7.51015 | 14237.33 | [M+H]+ | 86.3 |
| 8285 | (+)-Syringaresinol | 7.542316 | 17983.92 | [M+H]+ | 86.3 |
| 8353 | sugetriol | 7.670817 | 41759.84 | [M+H]+ | 84.8 |
| 8446 | [(3R)-3,7-dimethyloct-6-enyl] butanoate | 7.75515 | 23734.61 | [M+2H]2+ | 85.2 |
| 8483 | kaempferol | 7.775466 | 5663.169 | [M+H]+ | 86.6 |
| 8489 | apigenin-7-o-glucoside | 7.775466 | 48649.4 | [M+H]+ | 87.8 |
| 8515 | agrimonolide-6-o-β-d-glucopyranoside | 7.8093 | 43253.18 | [M+H]+ | 82.9 |
| 8540 | indene | 7.836633 | 9634.119 | [M+H]+ | 87.7 |
| 8642 | isorhamnetin | 7.986633 | 9591.763 | [M+H]+ | 86.9 |
| 8717 | procyanidin c1 | 8.068283 | 162337.9 | [M+H]+ | 86.7 |
| 8769 | Caffeate | 8.13095 | 19073.17 | [M+H]+ | 88.1 |
| 8805 | Khellol | 8.162117 | 5813.053 | [M+H]+ | 88.2 |
| 8890 | atractylenolide i | 8.290283 | 15467.3 | [M+H]+ | 87.2 |
| 8943 | alpha-cyperone | 8.304116 | 47127.82 | [M+H]+ | 87.9 |
| 9029 | liensinine | 8.385616 | 5610.315 | [M+H]+ | 80.7 |
| 9035 | astragaloside iii | 8.385616 | 10138.99 | [M+H]+ | 85.4 |
| 9136 | ()-Cuparene | 8.555767 | 13912.21 | [M+H]+ | 88.7 |
| 9244 | (3R)-3-(2-hydroxy-3,4-dimethoxyphenyl)chroman-7-ol | 8.703433 | 27968.97 | [M+H]+ | 88.5 |
| 9283 | Isohomogenol | 8.752934 | 13596.39 | [M+H]+ | 88.8 |
| 9309 | hederagenin | 8.793083 | 5416.241 | [M+H]+ | 88.7 |
| 9394 | flavonoids | 8.957084 | 5771.993 | [M+H]+ | 89.1 |
| 9410 | gamma-Calacorene | 8.99825 | 14140.58 | [M+H]+ | 88.7 |
| 9411 | Capsidiol | 8.99825 | 28616.66 | [M+H]+ | 89.1 |
| 9417 | sugeonyl acetate | 9.005584 | 16852.28 | [M+H]+ | 88.8 |
| 9443 | oxypaeoniflorin | 9.05475 | 14765.59 | [M+H]+ | 87.8 |
| 9513 | 2-(2-butynyl)-cyclohexanone | 9.134067 | 10994.79 | [M+H]+ | 89.2 |
| 9535 | isokobusone | 9.175567 | 38558.14 | [M+H]+ | 88.7 |
| 9552 | Teresautalic acid | 9.1919 | 14059.58 | [M+H]+ | 89.2 |
| 9564 | cycloastragenol | 9.199233 | 5687.417 | [M+H]+ | 88.8 |
| 9665 | pachypodol | 9.335067 | 41801.79 | [M+H]+ | 87.7 |
| 9668 | ononin | 9.335067 | 240720.9 | [M+H]+ | 89.4 |
| 9814 | (1R)-1-(4-methylphenyl)ethanol | 9.489233 | 13887.37 | [M+H]+ | 88.2 |
| 10052 | foliosidine | 9.9227 | 19737.59 | [M+H]+ | 86.4 |
| 10102 | (5r,7r,10s)-isopterocarpolon β-d-gluco-pyranoside | 10.00003 | 22897.32 | [M+H]+ | 89.1 |
| 10224 | gamma-nonalactone | 10.32285 | 48414.25 | [M+H]+ | 89 |
| 10290 | Cauloside A | 10.39935 | 6189.274 | [M+H]+ | 84.9 |
| 10291 | astramembrannin ii | 10.39935 | 23053.23 | [M+H]+ | 88.6 |
| 10464 | asernestioside A | 10.81767 | 4929.738 | [M+H]+ | 89.1 |
| 10494 | Khell | 10.93715 | 14430.7 | [M+H]+ | 90.2 |
| 10552 | eugenol | 11.06398 | 25067.74 | [M+H]+ | 90.2 |
| 10579 | Mairin | 11.08682 | 13451.87 | [M+H]+ | 91 |
| 10645 | Visnagin | 11.23598 | 9190.078 | [M+H]+ | 86.8 |
| 10754 | (+)-Syringaresinol-O-beta-D-glucoside | 11.5683 | 26723.19 | [M+H]+ | 91.4 |
| 10764 | Embelin | 11.62678 | 24859.92 | [M+H]+ | 91.3 |
| 10768 | 11-deoxyalisol a | 11.63462 | 14894.69 | [M+H]+ | 91.1 |
| 10807 | agrimophol | 11.76595 | 79180.41 | [M+H]+ | 84.4 |
| 10818 | emodin | 11.85928 | 15106.47 | [M+H]+ | 84.3 |
| 10992 | 2'-hydroxy-3',4'-dime thoxy-isoflavane-7-o-β-d-glucoside | 12.2506 | 8676.521 | [M+H]+ | 82 |
| 11007 | higenamine | 12.32208 | 10714.96 | [M+H]+ | 86.3 |
| 11059 | α-hederin | 12.45125 | 15365.76 | [M+H]+ | 89.8 |
| 11175 | (1S,3aR,4R,8aS)-7-isopropyl-1,4-dimethyl-2,3,3a,5,6,8a-hexahydroazulene-1,4-diol | 12.85307 | 32523.2 | [M+H]+ | 92.4 |
| 11201 | rosenonolactone | 12.90307 | 13233.06 | [M+H]+ | 87.4 |
| 11224 | poricoicacid g | 12.98507 | 14329.64 | [M+H]+ | 89 |
| 11283 | Quinicine | 13.14888 | 16282.16 | [M+H]+ | 80.5 |
| 11355 | 2,6-ditertbutyl-4methyl phenol | 13.5252 | 18993.01 | [M+H]+ | 84.6 |
| 11789 | Poricoic acid A | 14.55815 | 11817.4 | [M+H]+ | 92.2 |
| 11836 | Peraksine | 14.58082 | 8334.436 | [M+H]+ | 84 |
| 11867 | Hepta-3 | 14.59532 | 332185.4 | [M+H]+ | 91.8 |
| 11918 | Oroxindin | 14.62348 | 127139.6 | [M+H]+ | 93.8 |
| 12169 | lysine | 14.84565 | 11445.59 | [M+H]+ | 89.8 |
| 12290 | dehydroabieticacid methyl ester | 14.96013 | 4305.369 | [M+H]+ | 92.6 |
| 12376 | astragaloside v | 15.03197 | 45883.18 | [M+H]+ | 90.8 |
| 12411 | 16β-hydroxyalisol B monoacetate | 15.05463 | 8312.018 | [M+H]+ | 87 |
| 12426 | alisol a 24-acetate | 15.0618 | 8341.713 | [M+H]+ | 85 |
| 12509 | methyl thymyl ether | 15.13845 | 6803.525 | [M+H]+ | 93.7 |
| 12533 | l-tyrosine | 15.15245 | 11797.7 | [M+H]+ | 90.1 |
| 12750 | astrachrysoside A | 15.34895 | 5940.591 | [M+H]+ | 91.6 |
| 12837 | (?)-norrotundene | 15.41495 | 9848.896 | [M+H]+ | 91.3 |
| 12884 | benzoyl paeoniflorin | 15.45562 | 5679.541 | [M+H]+ | 94 |
| 12886 | prosapogenin cp2a | 15.45562 | 6889.208 | [M+H]+ | 94.4 |
| 12901 | decoyl vanillylamide | 15.46312 | 17534.16 | [M+H]+ | 83.5 |
| 12961 | embinin | 15.51077 | 7045.224 | [M+H]+ | 81.5 |
| 13300 | 13-hydroxy-9,11-octadecadienoic acid | 15.80543 | 9599.162 | [M+H]+ | 92.8 |
| 13325 | Poricoic acid B | 15.83243 | 29630.17 | [M+H]+ | 94.7 |
| 13343 | beta carotene | 15.84908 | 607550.4 | [M+H]+ | 81.6 |
| 13354 | astragalosideII | 15.84908 | 117187.8 | [M+H]+ | 94 |
| 13380 | 1,1,6-trimethyl-2H-naphthalene | 15.87208 | 31304.82 | [M+H]+ | 94.5 |
| 13405 | stigmast-4-ene-1,3-dione | 15.87208 | 5161.269 | [M+H]+ | 85.3 |
| 13426 | β-amyrin acetate | 15.88042 | 33315.29 | [M+H]+ | 80.3 |
| 13526 | pectolinarin | 15.96992 | 9673.987 | [M+H]+ | 93.3 |
| 13670 | alisol f | 16.02408 | 17865.76 | [M+H]+ | 93.7 |
| 13672 | 13β,17β-epoxyalisol a | 16.02408 | 55363.56 | [M+H]+ | 94.9 |
| 13801 | polydatin | 16.08342 | 9362.26 | [M+H]+ | 94.6 |
| 13820 | poricoic acid DM | 16.09942 | 15781.47 | [M+H]+ | 94.3 |
| 14001 | glyceride | 16.21173 | 7164.966 | [M+H]+ | 94.3 |
| 14009 | arjungenin | 16.21892 | 19911.62 | [M+H]+ | 92.6 |
| 14031 | digitalis glycoside | 16.26058 | 9536.487 | [M+H]+ | 94.9 |
| 14049 | 3-methoxycinnamaldehyde | 16.29373 | 96649.5 | [M+H]+ | 95 |
| 14053 | beta-ionone | 16.3104 | 18361.67 | [M+H]+ | 94.6 |
| 14056 | 1,4-Epoxy-16-hydroxyheneicos-1,3,12,14,18-pentaene | 16.3104 | 5576.033 | [M+H]+ | 94.6 |
| 14210 | alpha-chamigrene | 16.43957 | 10676.31 | [M+H]+ | 95.1 |
| 14261 | (2R)-2-[(5R,10S,13R,14R,16R,17R)-16-hydroxy-3-keto-4,4,10,13,14-pentamethyl-1,2,5,6,12,15,16,17-octahydrocyclopenta[a]phenanthren-17-yl]-5-isopropyl-hex-5-enoic acid | 16.49657 | 15505.86 | [M+H]+ | 94.7 |
| 14266 | soyasaponin 1 | 16.49657 | 16657.91 | [M+H]+ | 94.4 |
| 14540 | astragaloside viii | 16.61322 | 19850.58 | [M+H]+ | 84.8 |
| 14576 | (2R)-2-[(3S,5R,10S,13R,14R,16R,17R)-3,16-dihydroxy-4,4,10,13,14-pentamethyl-2,3,5,6,12,15,16,17-octahydro-1H-cyclopenta[a]phenanthren-17-yl]-5-isopropyl-hex-5-enoic acid | 16.62807 | 15125.33 | [M+H]+ | 95.2 |
| 14587 | alizexol b | 16.64238 | 160342.6 | [M+H]+ | 95.2 |
| 14608 | atractyloside e | 16.64922 | 11344.07 | [M+H]+ | 84.2 |
| 14671 | astragalosideI | 16.69938 | 31347.24 | [M+H]+ | 92.8 |
| 14730 | poricoic acid D | 16.73722 | 32508.94 | [M+H]+ | 95.2 |
| 14919 | Cadalin | 16.87355 | 26542.3 | [M+H]+ | 95.4 |
| 14940 | 2-[(1R,3S,4S)-3-isopropenyl-4-methyl-4-vinylcyclohexyl]propan-2-ol | 16.89672 | 21632.82 | [M+H]+ | 93.5 |
| 14943 | santalyl acetate | 16.89672 | 29256.92 | [M+H]+ | 95.4 |
| 14955 | ledane | 16.90522 | 3907.982 | [M+H]+ | 91.1 |
| 15388 | 3β-p-hydroxybenzoyldehydrotumulosicacid | 17.22987 | 6021.729 | [M+H]+ | 89.5 |
| 15724 | acetylastragaloside | 17.41102 | 4062.179 | [M+H]+ | 94 |
| 15820 | ()-2-Carene | 17.52468 | 27957.42 | [M+H]+ | 95.9 |
| 15914 | Sulfoorientalol B | 17.65333 | 9605.901 | [M+H]+ | 95.6 |
| 15962 | 25-hydroxy-3-epidehydrotumulosicacid | 17.69883 | 15303.99 | [M+H]+ | 92.4 |
| 16136 | geranylacetone | 17.7735 | 6784.624 | [M+H]+ | 95.9 |
| 16270 | trillin | 17.966 | 8392.554 | [M+H]+ | 96 |
| 16325 | Antioxidant No. 33 | 18.01765 | 18313.8 | [M+H]+ | 96.2 |
| 16332 | linolenic acid | 18.01765 | 17410.66 | [M+H]+ | 93.8 |
| 16382 | 3β-hydroxy-16α-acetoxy-lanosta-7,9(11),24-trien-21-oicacid | 18.03198 | 19619.79 | [M+H]+ | 81.6 |
| 16739 | flavonoid | 18.27598 | 5086.09 | [M+H]+ | 82.4 |
| 16795 | 4-hydroxy-2,6-dimethyl-6-(3,7-dimethyl-2,6-octadienyl)-8-(3-methyl-2-butenyl)-2h-1-ben-zopyran-5,7(3h,6h)-dione | 18.40763 | 31132.61 | [M+H]+ | 96.4 |
| 17036 | isobutyric acid | 18.82662 | 20863.3 | [M+H]+ | 94 |
| 17042 | 2-ethyl-3-hydroxyhexyl butyrate | 18.82662 | 142062.6 | [M+H]+ | 96 |
| 17050 | OYA | 18.83362 | 254037.2 | [M+H]+ | 95.6 |
| 17175 | 13β,17β-epoxyalisol a 24-acetate | 18.91828 | 15301.12 | [M+H]+ | 86.9 |
| 17239 | [(1S,3R)-1-[(2R)-3,3-dimethyloxiran-2-yl]-3-[(5R,8S,9S,10S,11S,14R)-11-hydroxy-4,4,8,10,14-pentamethyl-3-oxo-1,2,5,6,7,9,11,12,15,16-decahydrocyclopenta[a]phenanthren-17-yl]butyl] acetate | 18.98928 | 7454.778 | [M+H]+ | 92 |
| 17530 | Ostruthin | 19.3446 | 38924.11 | [M+H]+ | 96.9 |
| 17533 | Sulcatone | 19.3516 | 24528.42 | [M+H]+ | 96.7 |
| 17647 | EIC | 19.70758 | 10814.58 | [M+H]+ | 92.5 |
| 17668 | neferine | 19.79473 | 10981.07 | [M+H]+ | 82.8 |
| 17686 | 7,9(11)-dehydropachymic acid | 19.8384 | 32739.3 | [M+H]+ | 96.7 |
| 17687 | 16β-methoxyalisol B monoacetate | 19.8384 | 17567.58 | [M+H]+ | 97.2 |
| 17819 | butenolide b | 20.0224 | 13751.25 | [M+H]+ | 91.2 |
| 17865 | 1-monolinolein | 20.08807 | 4354.445 | [M+H]+ | 95.8 |
| 17937 | diosgenin | 20.20488 | 41596.1 | [M+H]+ | 92.8 |
| 18104 | eudesobovatol a | 20.34138 | 4640.517 | [M+H]+ | 92.7 |
| 18106 | zoomaric acid | 20.34838 | 6495.439 | [M+H]+ | 97.4 |
| 18189 | (2r,3r,5r,7r,10s)-atractyloside g 2-o-β-d-glucopyranoside | 20.48687 | 105533.7 | [M+H]+ | 88.4 |
| 18199 | Agrimol F | 20.49387 | 30633.38 | [M+H]+ | 83.5 |
| 18283 | tumulosic acid | 20.71837 | 4038.798 | [M+H]+ | 91.3 |
| 18368 | dehydroeburicoic acid | 20.97252 | 54254.3 | [M+H]+ | 97.6 |
| 18464 | Glycerol palmitate | 21.26917 | 5357.753 | [M+H]+ | 97.9 |
| 18485 | Chrysanthemaxanthin | 21.31483 | 20649.05 | [M+H]+ | 82.7 |
| 18551 | Cheilanthifoline | 21.39817 | 55448.8 | [M+H]+ | 87.7 |
| 18863 | DEP | 22.15563 | 51113.09 | [M+H]+ | 93.4 |
| 18964 | (1s,4s,5r,7r,10r)-11,14-dihydroxyguai-3-one11-o-β-d-glucopyranoside | 22.53528 | 81463.61 | [M+H]+ | 89.2 |
| 19162 | pachymic acid | 24.19253 | 79343.99 | [M+H]+ | 99 |
| 19170 | cerebroside | 24.27703 | 6341.144 | [M+H]+ | 98.6 |
| 19182 | farnesylacetone | 24.56568 | 4605.483 | [M+H]+ | 98 |
| 19195 | cyclosieversigenin | 24.7325 | 13827.87 | [M+H]+ | 98.6 |
| 19718 | alexandrin | 28.16002 | 67496.39 | [M+H]+ | 99.2 |

**Table S3**

Negative ion chromatography (TIC) of HTSJD based on UPLC-MS/MS.

| **PeakID** | **Title** | **RT (min)** | **Area** | **Adduct** | **Total score** |
| --- | --- | --- | --- | --- | --- |
| 312 | aspartic acid | 1.1818 | 12652.35 | [M-H]- | 81.2 |
| 323 | L- | 1.189467 | 6398.611 | [M-H]- | 80.9 |
| 334 | Crystal VI | 1.196467 | 53318.04 | [M-H]- | 81.3 |
| 335 | adeninenucleoside | 1.196467 | 7163.843 | [M-H]- | 81.3 |
| 379 | alpha-D-Galp-(1->6)-alpha-D-Galp-(1->6)-D-Glu | 1.210967 | 107263.5 | [M-H]- | 81.3 |
| 397 | homoserine | 1.218467 | 23351.04 | [M-H]- | 81.5 |
| 398 | Dimethyl L-malate | 1.218467 | 70703.69 | [M-H]- | 81.5 |
| 399 | RAM | 1.218467 | 41725.5 | [M-H]- | 81.5 |
| 405 | alpha-D-fructofuranose | 1.218467 | 47797.64 | [M-H]- | 81.6 |
| 408 | verbascose | 1.218467 | 35373.13 | [M-H]- | 81.4 |
| 445 | L-Milchsaeure | 1.232633 | 14896.5 | [M-H]- | 81.6 |
| 450 | inositol | 1.232633 | 1032790 | [M-H]- | 81.5 |
| 468 | sucrose | 1.246967 | 1747228 | [M-H]- | 81.6 |
| 526 | albiflorin | 1.2678 | 6661.447 | [M-H]- | 81.5 |
| 572 | quinic acid | 1.2888 | 194392 | [M-H]- | 81.5 |
| 598 | Arabinose,d | 1.2958 | 51416.46 | [M-H]- | 81.5 |
| 623 | D-Galacturonic acid, homopolymer | 1.303133 | 29584.81 | [M-H]- | 81.5 |
| 715 | ononin | 1.339133 | 50881.86 | [M-H]- | 80 |
| 724 | MLT | 1.353633 | 1514371 | [M-H]- | 81.4 |
| 775 | AKG | 1.411633 | 4528.524 | [M-H]- | 81.1 |
| 798 | vitamin c | 1.419467 | 24832.4 | [M-H]- | 80.6 |
| 806 | (-)-epicatechin-3-o-gallate | 1.419467 | 90381.16 | [M-H]- | 80.5 |
| 946 | 2-furancarboxylicacid | 1.498283 | 163722 | [M-H]- | 81.8 |
| 1452 | uridine | 1.867767 | 66351.41 | [M-H]- | 81.9 |
| 1728 | (+)-Catechin-5-O-glucoside | 2.008267 | 9315.292 | [M-H]- | 81.9 |
| 1853 | guanosine | 2.132267 | 110615.5 | [M-H]- | 82.3 |
| 1909 | l-leucine | 2.199433 | 18657.42 | [M-H]- | 82.5 |
| 1930 | 1-o-galloyl-glucose | 2.26725 | 63818.06 | [M-H]- | 81.9 |
| 1946 | Flavaxin | 2.301417 | 14840.14 | [M-H]- | 81.5 |
| 2060 | gallic acid | 2.624233 | 33633.82 | [M-H]- | 82.9 |
| 2092 | Geniposidic acid | 2.6754 | 15086.35 | [M-H]- | 82.8 |
| 2101 | catalpol | 2.726233 | 10765 | [M-H]- | 83.1 |
| 2111 | 7-O-methylisomucronulatol | 2.769733 | 55098.21 | [M-H]- | 81.3 |
| 2142 | BZM | 2.8289 | 24779.56 | [M-H]- | 80.9 |
| 2265 | 2-deoxy-d-ribono-1,4-lactone | 3.12805 | 15400.66 | [M-H]- | 83.4 |
| 2281 | Trimethyl citrate | 3.152717 | 10477 | [M-H]- | 82.6 |
| 2291 | HMF | 3.186883 | 11901.88 | [M-H]- | 83.4 |
| 2311 | n-butyl-β-D-fructopyranoside | 3.2642 | 5071.281 | [M-H]- | 83.4 |
| 2360 | alpha-Thymidine | 3.476533 | 53615.07 | [M-H]- | 83.8 |
| 2367 | Citromitin | 3.4857 | 103181.8 | [M-H]- | 81.2 |
| 2373 | polydatin | 3.493367 | 12539.5 | [M-H]- | 81 |
| 2486 | Dimethyl anthranilate | 3.762517 | 152133.2 | [M-H]- | 84.1 |
| 2540 | protocatechuic acid | 3.941017 | 7767.47 | [M-H]- | 84.3 |
| 2565 | atractulodin | 4.084667 | 8892.09 | [M-H]- | 80.9 |
| 2566 | vanillic acid | 4.118834 | 14620.11 | [M-H]- | 84 |
| 2621 | 8-Isopentenyl-kaempferol | 4.364817 | 8866.586 | [M-H]- | 80.2 |
| 2724 | Heriguard | 4.587817 | 96064.79 | [M-H]- | 84.2 |
| 2727 | vanillin | 4.59665 | 8396.591 | [M-H]- | 84.8 |
| 2747 | atsudaidai | 4.670483 | 8354.615 | [M-H]- | 84.7 |
| 2811 | aucubin | 4.720133 | 8985.251 | [M-H]- | 85 |
| 2842 | coixol | 4.795133 | 26340.15 | [M-H]- | 85.1 |
| 2860 | atractyloside i | 4.8283 | 4349.017 | [M-H]- | 84.3 |
| 2889 | 2-Acridinecarboxylic acid | 4.9033 | 4601.517 | [M-H]- | 83 |
| 3025 | acetic acid | 5.146616 | 4171.619 | [M-H]- | 85.5 |
| 3102 | Beta-Glucan | 5.227783 | 21713.46 | [M-H]- | 83 |
| 3104 | isoflavanone | 5.234617 | 3568.686 | [M-H]- | 80.2 |
| 3106 | ellagic acid | 5.234617 | 9383.032 | [M-H]- | 84.3 |
| 3136 | 10-epiatractyloside a | 5.28145 | 18659.52 | [M-H]- | 83.3 |
| 3226 | tryptophan | 5.359117 | 74762.74 | [M-H]- | 85.6 |
| 3233 | paeonolide | 5.368283 | 9911.312 | [M-H]- | 85.6 |
| 3262 | geniposide | 5.4356 | 60098.2 | [M-H]- | 84.9 |
| 3280 | caproic acid | 5.4506 | 5361.124 | [M-H]- | 85.5 |
| 3310 | apigenin-7-o-glucoside | 5.494767 | 7487.701 | [M-H]- | 80.1 |
| 3318 | suffruticoside a | 5.5106 | 10857.82 | [M-H]- | 84.4 |
| 3322 | (2 e)-2-decene-4,6-diyne-1,8-diol 8-o-β-d-apio-furanosyl-(1→6)-β-d-glucopyranoside | 5.517933 | 18211.05 | [M-H]- | 85.2 |
| 3414 | rutin | 5.6116 | 76796.79 | [M-H]- | 83.3 |
| 3462 | PHB | 5.650767 | 39799.05 | [M-H]- | 85.8 |
| 3486 | catechin | 5.680433 | 7101.833 | [M-H]- | 82.3 |
| 3623 | 2-hydroxy-3-methoxystrychnine | 5.82025 | 177709.8 | [M-H]- | 81.9 |
| 3667 | colchamine | 5.86275 | 3767.711 | [M-H]- | 84.5 |
| 3785 | 3'-Hydroxy-4'-methoxyisoflavone-7-O-beta-D-glucoside | 5.965583 | 16129.41 | [M-H]- | 85 |
| 3791 | FERULIC ACID (CIS) | 5.972417 | 5509.862 | [M-H]- | 86.2 |
| 3856 | icariside f2 | 6.01825 | 53931.89 | [M-H]- | 86.2 |
| 3900 | Caffeate | 6.05475 | 34414.32 | [M-H]- | 86.1 |
| 3984 | 2-hydroxy-4-methyl acetophenone | 6.1314 | 3280.15 | [M-H]- | 86.3 |
| 3986 | Flavonol | 6.1314 | 5990.374 | [M-H]- | 85.3 |
| 4117 | Plantaginin | 6.2139 | 6947.79 | [M-H]- | 86.3 |
| 4123 | ethyl gallate | 6.221567 | 11590.33 | [M-H]- | 86.3 |
| 4127 | rhamnocitrin-3-O-glucoside | 6.221567 | 34797.99 | [M-H]- | 85.2 |
| 4153 | caffeic acid dimethyl ether | 6.2374 | 13579.92 | [M-H]- | 85.4 |
| 4256 | narceine | 6.338567 | 34418.46 | [M-H]- | 83 |
| 4309 | Vanay | 6.405066 | 5132.257 | [M-H]- | 86.2 |
| 4423 | plantainoside D | 6.49005 | 19983.73 | [M-H]- | 82.5 |
| 4437 | atractylenolide i | 6.515883 | 4652.542 | [M-H]- | 80.4 |
| 4445 | naringin | 6.515883 | 22634.41 | [M-H]- | 85.2 |
| 4447 | syringin | 6.52355 | 22125.26 | [M-H]- | 86.4 |
| 4492 | benzoic acid | 6.600717 | 16131.42 | [M-H]- | 86.9 |
| 4508 | hellicoside | 6.608383 | 28313.01 | [M-H]- | 86.8 |
| 4539 | 25-hydroxy-3-epidehydrotumulosicacid | 6.63455 | 3831.508 | [M-H]- | 86.4 |
| 4641 | liensinine | 6.75055 | 10931.03 | [M-H]- | 82.4 |
| 4755 | elenolide | 6.849534 | 5592.882 | [M-H]- | 86 |
| 4826 | Martynoside | 6.930533 | 9982.954 | [M-H]- | 86.6 |
| 4889 | hesperidin methyl chalcone | 6.997366 | 40503.77 | [M-H]- | 84 |
| 4934 | embinin | 7.0502 | 8177.009 | [M-H]- | 80.7 |
| 4971 | calycosin | 7.0642 | 40171.5 | [M-H]- | 87.3 |
| 4996 | beta-elemene | 7.071367 | 4858.15 | [M-H]- | 85.5 |
| 4997 | cis-p-Coumarate | 7.071367 | 56665.46 | [M-H]- | 87.2 |
| 5022 | paeonol | 7.1127 | 10141.69 | [M-H]- | 86.9 |
| 5089 | atractylenolide iii | 7.20785 | 23609.06 | [M-H]- | 82.2 |
| 5115 | Ethyl glucoside | 7.224683 | 6883.72 | [M-H]- | 87.3 |
| 5155 | thebaine | 7.249017 | 4144.228 | [M-H]- | 87.4 |
| 5188 | 4-hydroxy-3,5-dimethoxyacetophenone | 7.281683 | 13977.75 | [M-H]- | 86.6 |
| 5342 | Cauloside A | 7.382017 | 6984.099 | [M-H]- | 87.5 |
| 5391 | 9,10-dimethoxypterocarpan-3-O-β-D-glucoside | 7.45085 | 8604.494 | [M-H]- | 87.4 |
| 5418 | (2S)-7-[(2S,3R,4S,5S,6R)-4,5-dihydroxy-6-methylol-3-[(2S,3R,4R,5R,6S)-3,4,5-trihydroxy-6-methyl-tetrahydropyran-2-yl]oxy-tetrahydropyran-2-yl]oxy-5-hydroxy-2-(3-hydroxy-5-methoxy-phenyl)chroman-4-one | 7.517833 | 27020.07 | [M-H]- | 80.2 |
| 5445 | rhamnocitrin | 7.54185 | 10445.02 | [M-H]- | 86.9 |
| 5457 | Calceolarioside A | 7.558517 | 9942.015 | [M-H]- | 87.3 |
| 5492 | anemonin | 7.608167 | 7778.29 | [M-H]- | 87.7 |
| 5505 | cinnamic acid | 7.625 | 4433.3 | [M-H]- | 87.8 |
| 5528 | khellol glucoside | 7.641333 | 12853.2 | [M-H]- | 87.9 |
| 5677 | galloylpaeoniflorin | 7.7205 | 14900.52 | [M-H]- | 87.9 |
| 5990 | epigallocatechin | 8.03865 | 6833.777 | [M-H]- | 87.4 |
| 6075 | 1,7-Dihydroxy-3,9-dimethoxy pterocarpene | 8.143817 | 5830.874 | [M-H]- | 88.1 |
| 6106 | benzoyl paeoniflorin | 8.178984 | 5507.219 | [M-H]- | 84.3 |
| 6407 | (+)-Syringaresinol | 8.4883 | 12219.99 | [M-H]- | 86.9 |
| 6410 | (+)-Syringaresinol-O-beta-D-glucoside | 8.496966 | 224273.9 | [M-H]- | 88.7 |
| 6474 | 5,4'-dihydroxyflavone-6-c-β-d-glycosyl-rhamnoside-7-o-glycoside | 8.60195 | 31363.83 | [M-H]- | 88.7 |
| 6479 | atractylon | 8.610784 | 4482.402 | [M-H]- | 83.5 |
| 6518 | Cheilanthifoline | 8.667283 | 8888.026 | [M-H]- | 82.7 |
| 6525 | Oroxindin | 8.676117 | 10469.31 | [M-H]- | 85.4 |
| 6538 | 2'-hydroxy-3',4'-dime thoxy-isoflavane-7-o-β-d-glucoside | 8.72495 | 6210.958 | [M-H]- | 86.7 |
| 6666 | 1beta-hydroxybaccatin i | 8.9706 | 726173.8 | [M-H]- | 88.5 |
| 6759 | Cleomin | 9.069767 | 8612.393 | [M-H]- | 86 |
| 6786 | sanguinarine | 9.111433 | 8706.668 | [M-H]- | 80.6 |
| 7193 | Sulcatone | 9.755733 | 21655.9 | [M-H]- | 88.8 |
| 7403 | 3,4-dihydroaucubin | 10.20855 | 5694.686 | [M-H]- | 90.2 |
| 7477 | vitexin xyloside | 10.32038 | 5886.809 | [M-H]- | 89.4 |
| 7507 | aucubigenin | 10.39403 | 754444.5 | [M-H]- | 90.4 |
| 7510 | emodin | 10.39403 | 7229.459 | [M-H]- | 90.3 |
| 7512 | cryptopine | 10.39403 | 8555.216 | [M-H]- | 80.3 |
| 7529 | pachypodol | 10.41505 | 73610.98 | [M-H]- | 89.2 |
| 7543 | gamma-aminobutyric acid | 10.45103 | 12446.8 | [M-H]- | 85.7 |
| 7756 | naringenin | 11.12683 | 6495.394 | [M-H]- | 90.8 |
| 7861 | (2r,3r,5r,7r,10s)-atractyloside g 2-o-β-d-glucopyranoside | 11.46667 | 9116.788 | [M-H]- | 90.7 |
| 7914 | Lariciresinol | 11.71932 | 8226.253 | [M-H]- | 88.7 |
| 7943 | 2'-O-beta-Glucopyranosyl-5,7,4',5'-tetrahydroxyflavanone | 11.82963 | 130272.1 | [M-H]- | 80.5 |
| 7950 | formononetin | 11.83665 | 56312.02 | [M-H]- | 91.5 |
| 7970 | 7,2'-dihydroxy-3',4'-dimethoxyisoflavone-7-O-β-D-glucoside | 11.8433 | 2461412 | [M-H]- | 91.6 |
| 8111 | DEP | 12.40395 | 9132.286 | [M-H]- | 91.4 |
| 8131 | neferine | 12.44928 | 20929.77 | [M-2H]2- | 86.4 |
| 8143 | 3β-[(o-β-d-glucopyranosyl-(1→4)-o-β-d-ribo-pyranosyl-(1→3)-o-α-l-rhamnopyranosyl-(1→2)-α-l-arabinopyranosyl)oxy]olean-12-en-28-oicacid o-β-d-glucopyranosyl ester | 12.45678 | 9246.895 | [M-H]- | 87.3 |
| 8165 | NO-biletin | 12.57793 | 13476.09 | [M-H]- | 92.1 |
| 8170 | Chrysanthemaxanthin | 12.5936 | 8131.655 | [M-H]- | 83.1 |
| 8227 | 3,5-dimethoxystilbene | 12.8266 | 16558.52 | [M-H]- | 90 |
| 8326 | tangeretin | 13.15258 | 52768.44 | [M-H]- | 92.7 |
| 8375 | agrimonolide-6-o-β-d-glucopyranoside | 13.4859 | 55826.46 | [M-H]- | 91.8 |
| 8547 | flavonoid | 14.0422 | 6741.23 | [M-H]- | 86.7 |
| 8755 | Agrimol C | 14.51602 | 22329.88 | [M-H]- | 90.5 |
| 8774 | (3R)-3-(2-hydroxy-3,4-dimethoxyphenyl)chroman-7-ol | 14.54318 | 6643.34 | [M-H]- | 89.7 |
| 8776 | 9,10-dimethoxy-pterocarpane-3-o-beta-d-glucoside | 14.54318 | 763434.3 | [M-H]- | 93.7 |
| 9059 | (1s,4s,5r,7r,10r)-11,14-dihydroxyguai-3-one11-o-β-d-glucopyranoside | 14.755 | 18933.48 | [M-H]- | 93.9 |
| 9082 | (6aR,11aR)-9,10-dimethoxy-6a,11a-dihydro-6H-benzofurano[3,2-c]chromen-3-ol | 14.77067 | 4097.411 | [M-H]- | 93.9 |
| 9436 | Dihydrocapsaicin | 15.06598 | 5236.266 | [M-H]- | 90 |
| 9458 | Ruvoside | 15.08832 | 8074.398 | [M-H]- | 94.2 |
| 9526 | astragaloside v | 15.12815 | 20462.18 | [M-H]- | 92.2 |
| 10217 | synepherine | 15.80028 | 3560.63 | [M-H]- | 94.6 |
| 10226 | 3-O-beta-D-Glucuronopyranosyl gypsogenin | 15.80028 | 10538.05 | [M-H]- | 93.8 |
| 10231 | atractyloside c | 15.80778 | 12575.24 | [M-H]- | 93.3 |
| 10278 | arjungenin | 15.86612 | 10200.14 | [M-H]- | 94.7 |
| 10339 | poricoicacid g | 15.87345 | 378440.4 | [M-H]- | 89.3 |
| 10341 | alisol f | 15.87345 | 17892.89 | [M-H]- | 94.6 |
| 10405 | Bifendate | 15.92978 | 12922.17 | [M-H]- | 93.9 |
| 10447 | 13β,17β-epoxyalisol a | 15.96278 | 11227.49 | [M-H]- | 94.7 |
| 10596 | α-hederin | 16.03277 | 41836.67 | [M-H]- | 90.6 |
| 10610 | (5r,7r,10s)-isopterocarpolon β-d-gluco-pyranoside | 16.03943 | 61942.86 | [M-H]- | 85.9 |
| 10681 | trillin | 16.07395 | 12382.84 | [M-H]- | 94.1 |
| 10731 | astragaloside iii | 16.11627 | 17494.65 | [M-H]- | 94.1 |
| 10886 | glyceride | 16.21577 | 14130.26 | [M-H]- | 94.9 |
| 10921 | sugetriol | 16.27127 | 109658 | [M-H]- | 94.9 |
| 10956 | foliosidine | 16.3296 | 12159.62 | [M-H]- | 85 |
| 10957 | Sulfoorientalol B | 16.3296 | 15740.46 | [M-H]- | 84.1 |
| 11102 | neryl acetate | 16.4381 | 6406.648 | [M-H]- | 95 |
| 11137 | acetylastragaloside | 16.47742 | 5053.363 | [M-H]- | 86.2 |
| 11263 | cis-isoascaridole | 16.56758 | 5742.85 | [M-H]- | 94.7 |
| 11330 | astragaloside viii | 16.61525 | 80007.59 | [M-H]- | 95.2 |
| 11345 | soyasaponin 1 | 16.62192 | 356062 | [M-H]- | 95.1 |
| 11482 | poricoic acid D | 16.73108 | 8483.097 | [M-H]- | 88.3 |
| 11508 | astragalosideII | 16.74623 | 31864.61 | [M-H]- | 95.3 |
| 11588 | limonin | 16.81973 | 97071.19 | [M-H]- | 95.2 |
| 11650 | atractyloside a 14-o-β-d-fructofuranoside | 16.86073 | 15373.06 | [M-H]- | 90.2 |
| 11748 | asernestioside B | 16.97623 | 7007.247 | [M-H]- | 90.9 |
| 11832 | Quinicine | 17.07207 | 27227.14 | [M-H]- | 80 |
| 11981 | Hyndarin | 17.20555 | 3403.327 | [M-H]- | 88.9 |
| 11995 | Antioxidant No. 33 | 17.21288 | 7431.35 | [M-H]- | 95.6 |
| 12142 | isokobusone | 17.34088 | 8921.319 | [M-H]- | 95.5 |
| 12204 | TR-saponin A | 17.41772 | 39336.23 | [M-H]- | 95.8 |
| 12311 | Poricoic acid B | 17.55503 | 8311.319 | [M-H]- | 91.2 |
| 12373 | alizexol b | 17.69353 | 17307.68 | [M-H]- | 95.7 |
| 12461 | astrasieversianin XV | 17.77003 | 7015.94 | [M-H]- | 92.9 |
| 12475 | Proantho Cyanidins | 17.80368 | 8318.492 | [M-H]- | 91.3 |
| 12481 | astragalosideI | 17.83152 | 7653.705 | [M-H]- | 95.6 |
| 12490 | Embelin | 17.85435 | 44032.87 | [M-H]- | 95.9 |
| 12516 | atractyloside b | 17.90852 | 39632.94 | [M-H]- | 90.4 |
| 12538 | Isohomogenol | 17.94002 | 5275.077 | [M-H]- | 95.9 |
| 12563 | prosapogenin cp2a | 17.96352 | 13504.92 | [M-H]- | 91.2 |
| 12761 | cycloastragenol | 18.20683 | 10675.47 | [M-H]- | 90.3 |
| 12867 | 4-hydroxy-2,6-dimethyl-6-(3,7-dimethyl-2,6-octadienyl)-8-(3-methyl-2-butenyl)-2h-1-ben-zopyran-5,7(3h,6h)-dione | 18.39767 | 5408.369 | [M-H]- | 96.4 |
| 12906 | Poricoic acid A | 18.43253 | 70983.5 | [M-H]- | 96.1 |
| 13104 | 3β-hydroxy-16α-acetoxy-lanosta-7,9(11),24-trien-21-oicacid | 18.84515 | 5229.331 | [M-H]- | 90.4 |
| 13114 | [(1S,3R)-1-[(2R)-3,3-dimethyloxiran-2-yl]-3-[(5R,8S,9S,10S,11S,14R)-11-hydroxy-4,4,8,10,14-pentamethyl-3-oxo-1,2,5,6,7,9,11,12,15,16-decahydrocyclopenta[a]phenanthren-17-yl]butyl] acetate | 18.91197 | 18873.89 | [M-H]- | 96.5 |
| 13116 | 13β,17β-epoxyalisol a 24-acetate | 18.91197 | 35936.96 | [M-H]- | 96.7 |
| 13227 | poricoic acid DM | 19.12147 | 11309.75 | [M-H]- | 96.8 |
| 13303 | 2-ethyl-3-hydroxyhexyl butyrate | 19.25078 | 7627.224 | [M-H]- | 96.5 |
| 13364 | hederagenin | 19.46595 | 9938.8 | [M-H]- | 96.6 |
| 13395 | orientalol e | 19.56828 | 12055.16 | [M-H]- | 97 |
| 13550 | Atractyloyne | 20.00892 | 8232.671 | [M-H]- | 96.9 |
| 13759 | (2R)-2-[(5R,10S,13R,14R,16R,17R)-16-hydroxy-3-keto-4,4,10,13,14-pentamethyl-1,2,5,6,12,15,16,17-octahydrocyclopenta[a]phenanthren-17-yl]-5-isopropyl-hex-5-enoic acid | 20.31857 | 83755.96 | [M-H]- | 97.2 |
| 13761 | (2R)-2-[(3S,5R,10S,13R,14R,16R,17R)-3,16-dihydroxy-4,4,10,13,14-pentamethyl-2,3,5,6,12,15,16,17-octahydro-1H-cyclopenta[a]phenanthren-17-yl]-5-isopropyl-hex-5-enoic acid | 20.31857 | 8552.751 | [M-H]- | 87.5 |
| 13774 | 16β-hydroxyalisol B monoacetate | 20.3474 | 91963.41 | [M-H]- | 96.5 |
| 13839 | 13-hydroxy-9,11-octadecadienoic acid | 20.4594 | 14407.65 | [M-H]- | 97.4 |
| 13841 | DIBP | 20.46607 | 15045.69 | [M-H]- | 97.2 |
| 13896 | decumbesterone A | 20.56823 | 8332.523 | [M-H]- | 85.2 |
| 13923 | Sulfoorientalol A | 20.5884 | 4805.527 | [M-H]- | 86 |
| 14027 | Capsidiol | 20.94238 | 37130.87 | [M-H]- | 97.7 |
| 14032 | tumulosic acid | 20.94972 | 247533.3 | [M-H]- | 97.7 |
| 14036 | alisol a 24-acetate | 20.94972 | 35553.26 | [M-H]- | 97.6 |
| 14052 | alpha-cyperone | 20.95655 | 29830.93 | [M-H]- | 97.1 |
| 14071 | 16β-methoxyalisol B monoacetate | 20.97772 | 31819.76 | [M-H]- | 96.5 |
| 14308 | ginsenoside rb1 | 21.48118 | 9982.288 | [M-H]- | 96.8 |
| 14368 | 7,9(11)-dehydropachymic acid | 21.59402 | 4901.803 | [M-H]- | 92.7 |
| 14458 | atractyloside e | 21.8475 | 9860.375 | [M-H]- | 98.1 |
| 14604 | Catharanthamine | 22.6558 | 8430.313 | [M-H]- | 92.8 |
| 14683 | pachymic acid | 23.81642 | 6604.942 | [M-H]- | 94.7 |
| 14716 | solamargine | 24.18955 | 11566.27 | [M-H]- | 94.8 |
| 14743 | linolenic acid | 24.37688 | 49845.18 | [M-H]- | 98.6 |
| 14775 | myristic acid | 24.87403 | 16956.06 | [M-H]- | 99.3 |
| 14787 | zoomaric acid | 25.15785 | 9167.241 | [M-H]- | 99 |
| 14788 | 3β-hydroxylanosta-7,9(11),24-trien-21-oic acid | 25.17818 | 5391.229 | [M-H]- | 96.9 |
| 14801 | 11-deoxyalisol a | 25.55883 | 5636.183 | [M-H]- | 91.6 |
| 14802 | oleic acid | 25.5655 | 19370.22 | [M-H]- | 85.8 |
| 14807 | EIC | 25.57217 | 749412.8 | [M-H]- | 99.5 |
| 14912 | Methyl myristate | 25.85382 | 10250 | [M-H]- | 99.5 |
| 14919 | Mairin | 26.05613 | 308828.3 | [M-H]- | 97.3 |
| 14959 | dehydroeburicoic acid | 26.37845 | 47417.03 | [M-H]- | 98.7 |
| 14979 | hexadecanoic acid | 26.7206 | 1055193 | [M-H]- | 99.4 |
| 15001 | Coixendide | 26.7271 | 6527.658 | [M-H]- | 94.1 |
| 15003 | 3beta-Hydroxy-24-methylene-8-lanostene-21-oic acid | 26.74027 | 39161.66 | [M-H]- | 96.7 |
| 15039 | methyl palmitate | 27.26658 | 11679.77 | [M-H]- | 99.7 |
| 15040 | 1-monolinolein | 27.26658 | 18321.17 | [M-H]- | 99.8 |
| 15047 | Glycerol palmitate | 28.30653 | 7701.375 | [M-H]- | 99.5 |
| 15051 | stearic acid | 28.3267 | 269377.8 | [M-H]- | 99.8 |

**Table S4**

Information on the Key Ingredients of HTSJD Actin on O-O

| **ID** | **PubChem CID** | **Key Ingredients** | **Degree** | **3D Structure** | **Corresponding Chinese herbs** |
| --- | --- | --- | --- | --- | --- |
| A1 | 10380176 | (3R)-7,2'-Dihydroxy-3',4'-dimethoxyisoflavan | 100 | 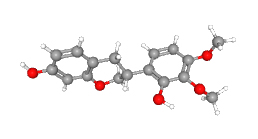 | Huangqi |
| A5 | 15689652 | 7-O-Methylisomucronulatol | 90 | 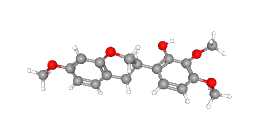 | Huangqi |
| A11 | 5280448 | Calycosin | 72 | 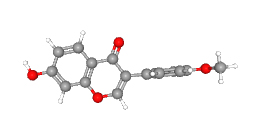 | Huangqi |
| A12 | 5280378 | Formononetin | 90 | 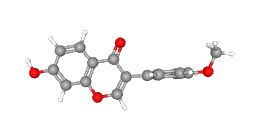 | Huangqi |
| A14 | 64971 | Mairin | 51 | 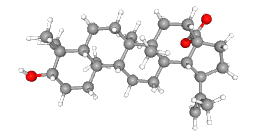 | Huangqi |
| A20 | 150893 | Hepta-3 | 111 | 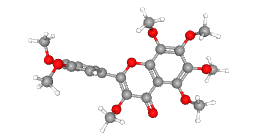 | Chenpi |
| A21 | 439246 | Naringenin | 121 | 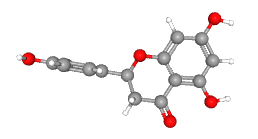 | Chenpi |
| A22 | 68077 | Tangeretin | 124 | 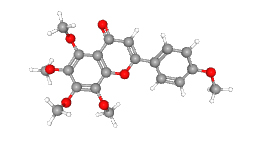 | Chenpi |
| A23 | 15225964 | Dehydrotumulosic Acid | 76 | 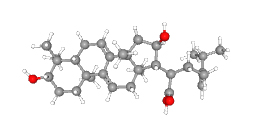 | Fuling |
| A24 | 9805290 | Polyporenic Acid C | 101 | 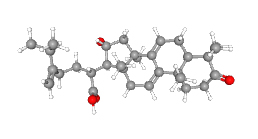 | Fuling |
| A25 | 73402 | Eburicoic acid | 93 | 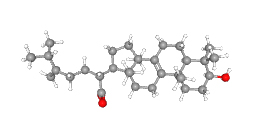 | Fuling |
| A26 | 15391340 | 3-Dehydrotrametenolic Acid | 65 | 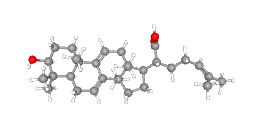 | Fuling |
| A28 | 15250826 | Dehydroeburicoic Acid | 69 | 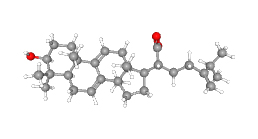 | Fuling |
| A29 | 5484385 | Pachymic Acid | 54 | 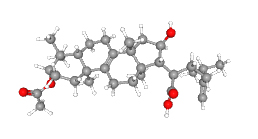 | Fuling |
| A34 | 12314446 | Tumulosic Acid | 97 | 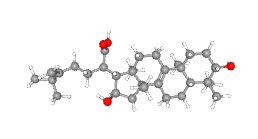 | Fuling |
| A35 | 10988340 | Alismoxide | 68 | 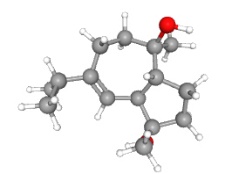 | Zexie |
| A36 | 14036811 | Alisol B 23-Acetate | 51 | 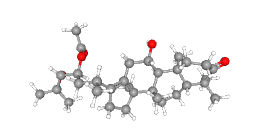 | Zexie |
| A40 | 3220 | Emodin | 73 | 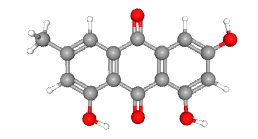 | Zexie |
| A44 | 78384888 | 17-(Furan-2-yl)heptadeca-3,7,9-trien-6-ol | 101 | 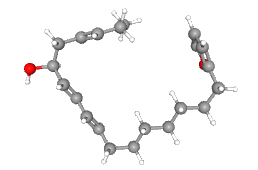 | Xiangfu |
| A45 | 5318624 | 8-Prenylkaempferol | 110 | 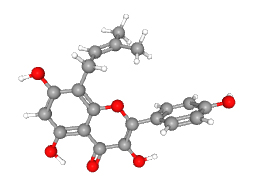 | Xiangfu |
| A47 | 72301 | Tetrahydropalmatine | 115 | 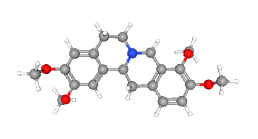 | Xiangfu |
| A48 | 3860435 | Isokobusone | 98 | 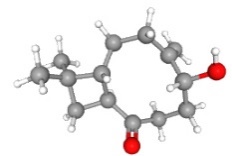 | Xiangfu |
| A52 | 11723309 | Rosenonolactone | 98 | 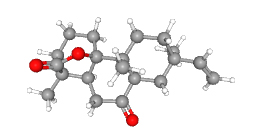 | Xiangfu |
| A53 | 46173924 | Sugeonyl Acetate | 105 | 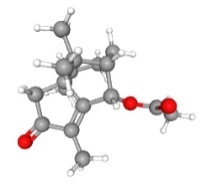 | Xiangfu |
| A60 | 99474 | Diosgenin | 52 | 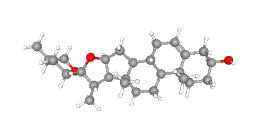 | Huangjing |
| B1 | 6603886 | Lopac-I-3766 | 101 | 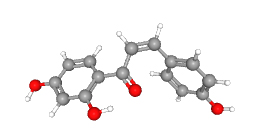 | Huangqi, Huangjing |
| B3 | 73299 | Hederagenin | 94 | 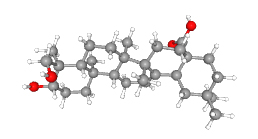 | Huangqi, Fuling |
| B4 | 5281654 | Isorhamnetin | 129 | 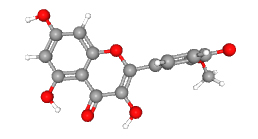 | Huangqi, Xiangfu |
| B5 | 5280863 | Kaempferol | 154 | 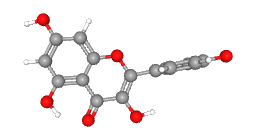 | Huangqi, Xiangfu |
| B6 | 5282822 | Elaidolinolenic acid | 110 | 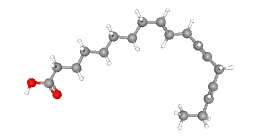 | Huangqi, Yiyiren |
| C1 | 5280343 | Quercetin | 226 | 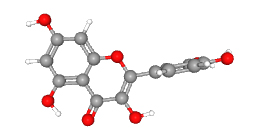 | Huangqi, Cheqianzi, Xiangfu |
| C2 | 445639 | Oleic Acid | 129 | 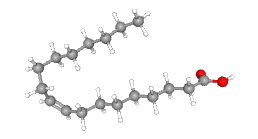 | Rougui, Sharen, Yiyiren |
| D1 | 5280450 | Linoleic acid | 104 | 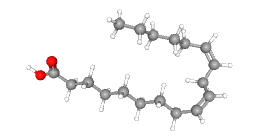 | Huangqi, Rougui, Sharen, Yiyiren |

**Table S5**

Affinity score and RMSD value of re-docking

| **Key Gene** | **Affinity** | **RMSD** |
| --- | --- | --- |
| EGFR | -7.9 | 0.742 (22 to 22 atoms) |
| IGF1R | -9.0 | 1.688 (37 to 37 atoms) |
| INSR | -8.6 | 1.053 (27 to 27 atoms) |
